# IL-4/IL-13-Driven Dysregulation of Epidermal Lipid Metabolism in Atopic Dermatitis: An Immunometabolic Link Between Type 2 Inflammation and Barrier Dysfunction

**DOI:** 10.3390/cells15121130

**Published:** 2026-06-22

**Authors:** Klara Andrzejczak, Agata Sternak, Wiktor Witkowski, Aleksandra Flak, Joanna Maj, Małgorzata Ponikowska

**Affiliations:** 1 Faculty of Medicine, Wroclaw Medical University, Wybrzeze Ludwika Pasteura 1, 50-367 Wroclaw, Poland; 2 University Centre of General Dermatology and Oncodermatology, Wroclaw Medical University, 50-556 Wroclaw, Poland; joanna.maj@umw.edu.pl

**Keywords:** atopic dermatitis, IL-4, IL-13, epidermal barrier, lipid metabolism, keratinocytes, JAK-STAT signaling, immunometabolism, ceramides, transepidermal water loss

## Abstract

Atopic dermatitis (AD) is a chronic immune-mediated inflammatory skin disease characterized by a complex and dynamic interplay between immune dysregulation and epidermal barrier dysfunction. Emerging evidence supports an integrated pathogenic model in which immune activation and barrier impairment form a bidirectional and self-reinforcing axis rather than representing separate processes. This review synthesizes current knowledge on the role of IL-4/IL-13-dependent signaling in regulating keratinocyte lipid metabolism and its impact on epidermal barrier integrity. IL-4/IL-13 signaling via the JAK-STAT pathway, particularly STAT6, contributes to keratinocyte dysfunction, resulting in impaired differentiation and coordinated alterations in lipid metabolism, including fatty acid elongation and ceramide synthesis. These cytokine-driven processes disrupt the organization of the stratum corneum lipid matrix, resulting in increased transepidermal water loss, enhanced skin permeability, and susceptibility to microbial colonization, thereby promoting chronic inflammation. Collectively, these findings support the concept that IL-4/IL-13-mediated dysregulation of keratinocyte lipid metabolism may represent an important immunometabolic mechanism linking type 2 inflammation with secondary barrier dysfunction in atopic dermatitis, thereby contributing to disease persistence. Targeting both immune pathways and epidermal lipid homeostasis may represent an effective strategy to restore barrier function and improve clinical outcomes.

## 1. Introduction

Atopic dermatitis (AD) is a chronic, relapsing, immune-mediated inflammatory skin disease characterized by intense pruritus, xerosis, and eczematous lesions. It is among the most prevalent inflammatory dermatoses, with disease onset typically occurring in early childhood. The clinical course is heterogeneous, with some patients achieving remission, while in others the disease persists or recurs into adulthood. This heterogeneity is also reflected in distinct clinical and immunological endotypes, including intrinsic and extrinsic AD, age-related differences between pediatric and adult disease, and ethnic variation in dominant immune signatures [1,2,3,4].

AD is often considered the first stage of the atopic march, characterized by the sequential development of allergic diseases such as food allergy, allergic rhinitis, and bronchial asthma, highlighting its systemic nature [1,5,6].

The pathogenesis of AD is multifactorial and involves complex interactions between genetic predisposition, immune dysregulation, and epidermal barrier dysfunction. Traditionally, the disease has been explained by the “outside-in” model, in which primary defects in the epidermal barrier, including alterations in structural proteins such as filaggrin, initiate immune activation. However, this concept does not fully explain disease heterogeneity or the presence of significant barrier dysfunction in patients without identifiable genetic mutations.

In the “inside-out” model, AD is a cytokine-driven disease in which inflammation leads to secondary impairment of the epidermal barrier. The immune response is predominantly driven by Th2 pathways, with IL-4 and IL-13 playing key roles.

AD pathogenesis is further influenced by additional mechanisms, including skin microbiota dysbiosis characterized by reduced diversity and overgrowth of *Staphylococcus aureus* and *Malassezia*, as well as an IgE-dependent immune response, all of which contribute to the persistence of chronic inflammation [6,7,8,9,10,11,12,13,14,15].

Keratinocytes play a key role in maintaining barrier integrity and coordinating immune responses. Increasing evidence indicates that type 2 cytokines not only impair keratinocyte differentiation but also directly influence lipid metabolic pathways essential for barrier formation.

Although metabolic reprogramming has been described in chronic inflammatory diseases, the relevance of this concept to epidermal lipid metabolism remains incompletely understood. In particular, it is unclear to what extent epidermal barrier dysfunction and abnormalities in lipid metabolism result from cytokine-induced changes that may be consistent with the concept of metabolic reprogramming in keratinocyte lipid pathways. Importantly, it remains to be determined whether these alterations represent a form of coordinated metabolic reprogramming or rather reflect broader metabolic dysregulation, highlighting the need for further mechanistic studies [16,17,18,19,20].

The epidermis, particularly the stratum corneum, plays a central role in this process as a key component of the skin barrier, with its properties largely determined by lipid composition and organization [14,17,21,22,23].

Current concepts of AD pathogenesis integrate the “outside-in” and “inside-out” models, highlighting a positive feedback loop in which barrier disruption promotes immune activation, while inflammatory cytokines further exacerbate barrier dysfunction [12,24,25]. These lipid alterations may contribute to increased transepidermal water loss (TEWL) and impaired epidermal barrier function [17].

Despite substantial progress in understanding AD pathogenesis, the mechanistic relationship between IL-4/IL-13 signaling and epidermal lipid metabolism remains incompletely defined, as evidence from experimental, lipidomic, and clinical studies is often fragmented and focused on isolated aspects rather than a unified pathogenic pathway.

Previous reviews have extensively addressed type 2 inflammation, epidermal barrier dysfunction, and the broader immunopathogenesis of AD. However, the specific role of IL-4/IL-13-driven alterations in keratinocyte lipid metabolism as a mechanistic link between inflammation and secondary barrier impairment has not been comprehensively synthesized. To address this gap, this review integrates mechanistic, emerging omics, and clinical evidence to examine the concept that IL-4/IL-13-mediated dysregulation of keratinocyte lipid metabolism may represent an important immunometabolic link between type 2 inflammation and epidermal barrier dysfunction in atopic dermatitis. The available evidence is critically considered with attention to both its strengths and limitations, while recognizing that this pathway does not fully account for disease heterogeneity. Nevertheless, current data suggest that it is a significant contributor to barrier impairment.

This framework further links cytokine-driven lipid dysregulation with microbial dysbiosis and chronic inflammation, highlighting secondary barrier dysfunction as an important component of AD pathogenesis.

## 2. Epidermal Lipid Homeostasis and Barrier Formation

### 2.1. Structural Organization and Composition of the Epidermal Lipid Barrier

**Figure 1:**
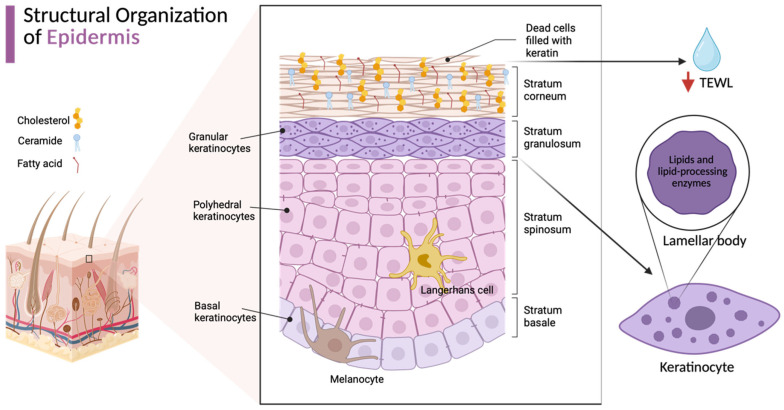
Structural Organization of the Epidermis and the Stratum Corneum Lipid Matrix. Created in BioRender. Andrzejczak, K. (2026) https://BioRender.com/yo0eotu. Abbreviations: TEWL, transepidermal water loss.

As the body’s largest organ, the skin functions as a critical protective barrier separating the body from the external environment. Its barrier properties are primarily localized to the stratum corneum (SC), the outermost layer of the epidermis (Figure 1) [22,26].

Structurally, the stratum corneum is organized in a brick-and-mortar arrangement, in which corneocytes, anucleate cells derived from keratinocytes and linked by corneodesmosomes, constitute the “bricks”, while the surrounding extracellular lipid matrix forms the “mortar” and represents a key component of the epidermal barrier.

This matrix is hydrophobic and consists primarily of ceramides (~50%), cholesterol (~25%), and free fatty acids (~15%); functionally, these lipids are present in an approximately equimolar ratio (~1:1:1). Unlike other biological membranes, the stratum corneum contains virtually no phospholipids, which confers unique structural and functional properties. These lipids form highly ordered, three-dimensional lamellar structures with specific lateral packing and lamellar organization, which are responsible for limiting transepidermal water loss and preventing the penetration of exogenous substances into the deeper layers of the skin. The proper organization and proportions of these lipids are crucial for maintaining the integrity of the epidermal barrier [15,22,26,27,28,29,30].

During terminal differentiation, keratinocytes migrate from the basal layer toward the epidermal surface, passing through the spinous and granular layers before reaching the stratum corneum, where they differentiate into corneocytes. This process is regulated by hormonal factors, cytokines, and environmental stimuli such as UV radiation. Granular layer keratinocytes play a key role, synthesizing epidermal barrier lipids and producing specialized organelles, lamellar bodies, which contain both lipids (including cholesterol, phospholipids, glycosylceramides, and sphingomyelin) and enzymes required for their further processing. These are then secreted into the intercellular space of the stratum corneum, where they undergo enzymatic processing, resulting in the formation of mature epidermal barrier lipids that organize into highly ordered lamellar structures [14,21,28,29,31,32,33].

The barrier function of the epidermis is further supported by tight junctions (TJs), located primarily in the granular layer and composed of claudins (including claudin-1) and occludin. These structures regulate the movement of water and electrolytes and interact with the lipid matrix to maintain barrier integrity [34].

Disturbances in epidermal barrier integrity, observed in atopic dermatitis in both affected and apparently unaffected skin, are characterized by alterations in lipid composition, increased TEWL, elevated pH, enhanced epidermal permeability, reduced stratum corneum hydration (SCH), and increased susceptibility to infections [15,35,36,37].

### 2.2. Ceramide Composition and VLCFAs (Very-Long-Chain Fatty Acids) in Epidermal Barrier Integrity

Ceramides (CER) are a key class of stratum corneum lipids and act synergistically with cholesterol and free fatty acids to form and maintain the epidermal barrier. Their functional properties largely depend on fatty acid composition and chain length. Ceramide biosynthesis occurs primarily in keratinocytes and involves a series of enzymatic steps.

Ceramides are composed of a sphingoid base linked to a fatty acid via an amide bond, and their structural diversity is determined by chain length, the degree of unsaturation, and the presence and position of hydroxyl groups. In the stratum corneum, four main types of sphingoid long-chain bases (LCB) can be distinguished: dihydrosphingosine (DS, sphinganine), sphingosine (S), phytosphingosine (P), and 6-hydroxysphingosine (H), as well as three types of fatty acids: non-hydroxy acids (N), α-hydroxy acids (A), and esterified ω-hydroxy acids (EO) [29,38,39].

Depending on carbon chain length, ceramides are classified as long-chain (approximately C14–C18/C20), very long-chain (approximately C20–C26), and ultra-long-chain (>C26), although these boundaries may vary depending on the source [39,40].

More than 20 ceramide subclasses have been identified in the human stratum corneum, differing in the type of sphingoid base as well as fatty acid chain length and structure [41].

Ceramide composition significantly influences the organization of lamellar structures and skin barrier properties, including regulation of TEWL, and is associated with the severity of atopic dermatitis. Even in the early stages of atopic dermatitis, changes in lipid composition occur in both affected and apparently unaffected skin. These include a reduction in the proportion of ceramides containing long- and very long-chain fatty acids, as well as a shift in the lipid profile toward shorter chains.

Shortening of fatty acid chains is associated with reduced lipid order and packing density within lamellar structures, resulting in increased epidermal barrier permeability. Of particular importance is the reduced proportion of very long-chain fatty acids, especially those around C24, in lesional skin. These changes are accompanied by altered proportions of ceramide subclasses, leading to disorganization of lamellar structures and impaired epidermal barrier integrity [27,38,39,42,43,44,45,46,47].

## 3. The Th2 Axis as a Key Pathogenic Mechanism in Atopic Dermatitis

### 3.1. The Th2 Inflammatory Environment and Epidermal Barrier Dysfunction

A characteristic immunological feature of atopic dermatitis is the dominance of type 2 inflammation, involving the activation of multiple immune cell populations, including Th2 lymphocytes and type 2 innate lymphoid cells (ILC2). IL-4 and IL-13 are central mediators of this response and contribute to chronic inflammation and disease exacerbations [48,49,50]. Polymorphisms in genes encoding IL-4, IL-13, and their receptors have also been associated with increased susceptibility to AD in both children and adults.

IL-4 plays an important role in initiating the type 2 immune response by promoting differentiation of naïve CD4+ T cells into Th2 cells, which subsequently produce IL-4, IL-13, and other type 2 mediators, thereby reinforcing the inflammatory loop [51,52,53].

Consistent with this pathogenic role, Th2 axis activation is associated with increased expression of IL-4 and IL-13 in both acute and chronic AD lesions, together with an increased frequency of Th2 cells in the skin of patients with moderate-to-severe disease compared with healthy individuals [53,54].

In type 2 inflammation, cytokines IL-4 and IL-13 affect keratinocytes, disrupting their differentiation and stratum corneum formation. Simultaneously, they inhibit the expression of key epidermal barrier proteins, such as filaggrin (FLG), loricrin (LOR), and involucrin (IVL). Reduced FLG levels may also indirectly influence lipid organization in lamellar bodies, underscoring the close interplay between keratinocyte differentiation and epidermal lipid metabolism.

IL-4 and IL-13 have also been shown to influence epidermal lipid metabolism by modulating pathways involved in lipid synthesis and processing. These effects may contribute to impaired stratum corneum organization and increased barrier vulnerability, which are explored in greater detail in Section 4 and Section 5. Additionally, IL-4 and IL-13 inhibit the expression of antimicrobial peptides (AMPs), such as β-defensins and cathelicidin LL-37, thereby increasing susceptibility to infection.

Disruption of epidermal barrier integrity leads to keratinocyte activation and initiation of inflammation. In response to barrier damage, epidermal cells release epithelial mediators that promote activation of the type 2 response and its further amplification [2,6,11,12,17,21,52,53,55,56,57,58,59].

In addition to the dominant Th2 axis, other immunological pathways, including Th17, Th22, and Th1, also contribute to AD pathogenesis and may further modulate epidermal barrier function through the action of IL-22 and IL-17A [48,58].

### 3.2. IL-4/IL-13 Receptor-Mediated Signaling Pathways and JAK-STAT Activation

IL-4 and IL-13 play a key role in the pathogenesis of atopic dermatitis by activating the intracellular signaling pathway involving Janus kinases (JAKs) and signal transducers and activators of transcription (STATs). The JAK family of kinases includes JAK1, JAK2, JAK3, and TYK2, while the STAT family consists of seven proteins, among which STAT6, and to a lesser extent STAT3, play key roles in the type 2 inflammatory response. The JAK-STAT pathway mediates signal transduction from the plasma membrane to the nucleus and is engaged by multiple cytokines.

When IL-4 and IL-13 bind to their respective cell surface receptors, JAKs are subsequently activated, leading to phosphorylation of STATs. Activated STATs regulate the expression of genes involved in the type 2 immune response and epidermal barrier function, including pathways involved in lipid metabolism. STAT6 plays a particularly important role as the primary effector of IL-4/IL-13 signaling, regulating the expression of genes involved not only in the type 2 immune response and keratinocyte differentiation, but also in the biosynthesis and organization of epidermal lipids [58,60,61,62].

IL-4 signaling is initiated by its binding to the IL-4Rα receptor subunit. The IL-4/IL-4Rα complex can form a functional receptor through association with one of two chains: the common γc chain (IL-2Rγc), forming a type I receptor, or IL-13Rα1, forming a type II receptor. IL-4 signals through both of these heterodimeric receptors, whereas IL-13 exclusively utilizes the IL-4Rα/IL-13Rα1 complex [52,63,64]. Despite the low affinity of IL-13Rα1 for its ligand, its association with IL-4Rα enables effective proinflammatory signaling.

Keratinocytes also express IL-13Rα2, which primarily functions as a decoy receptor, limiting IL-13 signaling and contributing to negative regulation [58,63,65,66]. However, emerging evidence suggests that IL-13Rα2 may also be involved in signal transduction, including through activation of pathways leading to increased TGF-β1 production [67,68].

The nature of signaling depends on cell type. In lymphocytes and dendritic cells, IL-4 signals through the IL-4Rα/γc complex, activating the kinases JAK1 and JAK3 and the transcription factor STAT6. In keratinocytes, which are non-hematopoietic cells, γc expression is low or absent, making IL-4Rα/IL-13Rα1 the dominant receptor complex. IL-4 and IL-13 activate this receptor, leading to activation of JAK1, JAK2, and TYK2, as well as the transcription factors STAT6 and STAT3.

**Figure 2:**
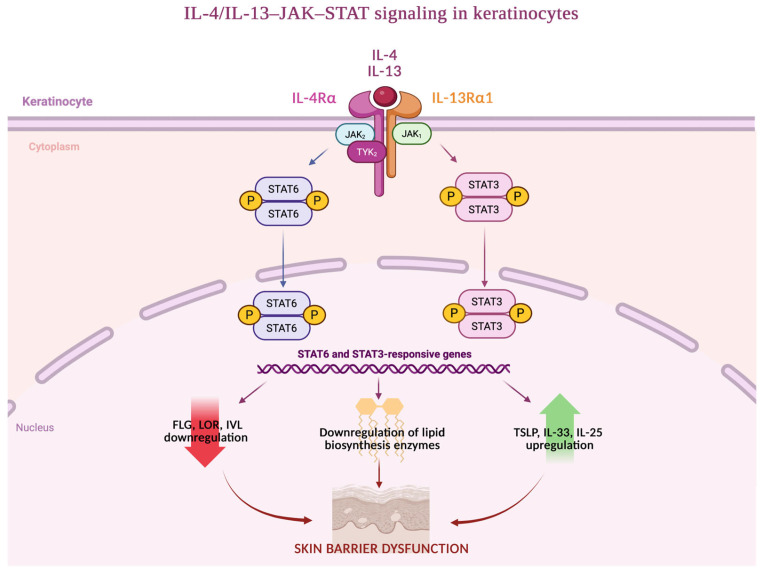
IL-4/IL-13–Induced JAK-STAT Signaling in Keratinocytes and Its Effects on Epidermal Barrier Function. Created in BioRender. Andrzejczak, K. (2026) https://BioRender.com/kvq4fw8. Abbreviations: FLG, filaggrin; IL, interleukin; IL-4Rα, interleukin-4 receptor alpha subunit; IL-13Rα1, interleukin-13 receptor alpha 1; IVL, involucrin; JAK, Janus kinase; LOR, loricrin; P, phosphorylation; STAT, signal transducer and activator of transcription; TSLP, thymic stromal lymphopoietin; TYK2, tyrosine kinase 2.

Activation of the JAK-STAT pathway in keratinocytes leads to disruption of keratinocyte differentiation, reduced expression of key epidermal barrier proteins, and dysregulation of epidermal lipid metabolism, resulting in secondary barrier dysfunction and promotion of chronic inflammation. These cytokine-driven mechanisms and their downstream effects on keratinocyte function are illustrated in Figure 2 [58,60,62,63,67,69].

Collectively, available data indicate that IL-4/IL-13 signaling via the JAK-STAT pathway, particularly involving STAT6, plays a key role in epidermal barrier dysfunction. This process involves impaired keratinocyte differentiation and dysregulation of epidermal lipid metabolism, including suppression of lipidogenic enzyme expression and disruption of the stratum corneum lipid matrix. Notably, alterations in key epidermal lipids, including ceramides, free fatty acids, and cholesterol, appear to represent a secondary consequence of cytokine-driven inflammation rather than solely a primary defect.

Supported by experimental, transcriptomic, and in vivo evidence, these findings indicate that Th2-dependent signaling induces coordinated metabolic alterations in keratinocytes, thereby contributing to barrier dysfunction and positioning secondary barrier impairment as a key component of AD pathogenesis within an integrated model extending beyond the traditional “outside-in” paradigm [14,17,25,39,60,70,71,72,73].

### 3.3. Keratinocytes as Amplifiers of Type 2 Inflammation

In addition to their structural function, keratinocytes play an active role in regulating skin immune responses. In the context of barrier disruption, their dysfunction can promote the progression of chronic inflammatory skin diseases. Under these conditions, keratinocytes promote a shift toward type 2 immunity and are considered key effector cells driving aberrant immune responses in atopic dermatitis.

In response to epidermal barrier damage and proinflammatory cytokines, particularly those associated with the type 2 immune response, such as IL-4 and IL-13, keratinocytes increase the production of epithelial alarmins, such as interleukin-25 (IL-25), interleukin-33 (IL-33), and thymic stromal lymphopoietin (TSLP), as well as other inflammatory mediators, including interleukin-1β (IL-1β). These mediators play a key role in activating dendritic cells and type 2 innate lymphoid cells (ILC2), leading to enhanced type 2 immune responses and increased production of cytokines characteristic of this pathway, including IL-4, IL-5, IL-13, and IL-31.

As a result, keratinocytes amplify the type 2 response by producing alarmins and inflammatory mediators, while type 2 cytokines further exacerbate barrier dysfunction through disruption of lipid metabolism and keratinocyte differentiation. This mechanism contributes to the persistence of chronic inflammation and disease progression.

In this model, keratinocytes are not merely passive targets of cytokine signaling, but active contributors to disease pathogenesis, functioning as active integrators of type 2 immune signaling, epidermal lipid dysregulation, and barrier dysfunction, thereby sustaining a self-amplifying inflammatory loop in atopic dermatitis. This cycle is further reinforced by lipid-mediated barrier dysfunction and microbial dysbiosis, forming an integrated pathogenic network [14,17,19,74,75,76].

## 4. Molecular Mechanisms of Lipid Suppression

### 4.1. Dysregulation of Fatty Acid Elongation and Ceramide Biosynthesis in Atopic Dermatitis

Various ceramide subclasses are present in the epidermis, among which skin-specific ceramides, such as EOS and EOP, play a particularly important role in maintaining barrier function. These ceramides are characterized by the presence of very-long and particularly ultra-long-chain fatty acids, which are essential for proper lamellar organization and epidermal barrier integrity [17,77,78].

In particular, ω-O-acylceramides, especially the EOS and EOP subclasses, are regarded as essential structural lipids of the stratum corneum. These ceramides contain ultra-long-chain ω-hydroxy fatty acids that are further esterified with linoleic acid, enabling the formation of the corneocyte lipid envelope (CLE) and proper organization of extracellular lamellar membranes. The CLE provides a covalently bound lipid scaffold surrounding corneocytes and plays a critical role in epidermal barrier stability and permeability regulation [79,80].

Proper epidermal barrier architecture further depends on the highly organized extracellular arrangement of ceramides, cholesterol, and free fatty acids within lamellar membranes. Altered cholesterol composition and disturbed cholesterol-to-ceramide ratios may impair lamellar membrane stability and contribute to defective barrier permeability and increased TEWL in AD [21,81].

Alterations in EOS and EOP ceramides have been repeatedly associated with impaired barrier function in AD. Reduced levels of ω-O-acylceramides and shortening of their fatty acid chains may impair lamellar membrane organization and contribute to increased TEWL. Experimental and translational studies suggest that disturbed synthesis of ultra-long-chain fatty acids, resulting from altered ELOVL and CerS3 activity, may contribute to defective ω-O-acylceramide production in AD [17,27,82]. The main structural and functional features of EOS and EOP ceramides are summarized in Table 1.

The biosynthesis of very-long-chain and ultra-long-chain fatty acyl-CoAs, which are incorporated into ceramides, occurs in two main stages. The first involves the synthesis of long-chain fatty acids (up to C16), followed by their elongation in the endoplasmic reticulum by a family of elongases known as very-long-chain 3-oxoacyl-CoA synthases (ELOVL1–7), each characterized by distinct substrate specificity. Among these, ELOVL1, ELOVL3, ELOVL4, and ELOVL6 appear to play particularly important roles in epidermal lipid homeostasis and are dysregulated in atopic dermatitis. The coordinated activity of these enzymes is important for maintaining proper lipid composition and epidermal barrier function [39,83].

In this context, elevated levels of IL-4 and IL-13 have been associated with downregulation of fatty acid elongases ELOVL3 and ELOVL6 in keratinocytes [17,77]. This may contribute to impaired fatty acid elongation and reduced synthesis of very-long-chain fatty acids (VLCFAs), despite compensatory changes in other elongases, including ELOVL1 and ELOVL4, which are also involved in epidermal fatty acid elongation [17,21].

Experimental studies in murine models and cultured human keratinocytes have shown that IL-4 and IL-13 can downregulate ELOVL3 and ELOVL6 expression in a STAT6-dependent manner, which has been associated with shortening of epidermal lipid chain length [14]. Consequently, patients with atopic dermatitis exhibit a reduced proportion of ceramides containing long-chain fatty acids and a relative increase in shorter-chain ceramides. These alterations in epidermal lipid composition are thought to contribute to lamellar disorganization, impaired barrier function, and increased TEWL [17,21,22,46].

Moreover, patients with atopic dermatitis exhibit reduced total ceramide levels compared to healthy individuals. Ceramides are synthesized in the epidermis by ceramide synthases (CerS), which catalyze the N-acylation of sphingoid bases with fatty acyl-CoAs. Six CerS isoforms (CerS1–CerS6) have been identified, each characterized by distinct substrate specificity with respect to acyl-CoA chain length. Importantly, reduced expression of specific isoforms, particularly CerS1 and CerS3, has been reported in atopic dermatitis [77,83].

Inhibition of ceramide synthase activity leads to significant disturbances in the lipid composition of the stratum corneum. Among these enzymes, CerS3 is of particular importance, as it plays a central role in the synthesis of ceramides containing ultra-long acyl chains (ULC-Cers) and is highly expressed in keratinocytes. Ceramides generated by CerS3 are essential for maintaining epidermal barrier integrity and regulating water permeability, as they represent key components of the extracellular lipid lamellae [82,84].

Animal studies have demonstrated that CerS3 deficiency results in a near-complete loss of ceramides containing ultra-long-chain fatty acids, disruption of lipid lamellar organization, and increased TEWL [82]. Reduced CerS3 expression has been associated with impaired epidermal barrier function and may contribute to the persistence of chronic inflammation, supporting a link between lipid metabolic dysregulation and immune responses in AD [77,83]. However, although experimental and translational studies support these mechanistic relationships, direct causal evidence confirming these pathways in humans remains incomplete. The major lipid abnormalities contributing to epidermal barrier dysfunction in AD are summarized in Table 2.

### 4.2. Transcriptional and Metabolic Regulation of Epidermal Lipid Homeostasis

#### 4.2.1. STAT6-Mediated Suppression of Lipid Metabolism

STAT6, a key mediator of IL-4/IL-13 signaling, acts as a central transcriptional regulator in keratinocytes. In a Th2 cytokine-dominated environment, its activation has been associated with changes in keratinocyte gene expression, including pathways involved in fatty acid elongation and ceramide synthesis [14,21].

These observations are supported by data indicating that IL-4/IL-13-mediated suppression of ELOVL elongases may be STAT6-dependent, and that inhibition of this pathway attenuates this effect, highlighting its role in the regulation of epidermal lipid metabolism [17].

Collectively, Th2 cytokine-driven STAT6 activation represents an important mechanism linking immune dysregulation with altered epidermal lipid metabolism and barrier dysfunction in AD. Through the modulation of genes involved in fatty acid elongation and keratinocyte differentiation, STAT6 may contribute to these processes [14,17,21,89].

#### 4.2.2. Nuclear Receptors in the Regulation of Epidermal Lipid Synthesis: PPAR-α, LXR and SREBP-1

The role of STAT6 in regulating lipid pathways has also been supported by findings from other cellular models. In murine hepatocytes, IL-4, acting through STAT6, was shown to inhibit the transcriptional activity of PPAR-α and impair its recruitment to promoter regions of target genes involved in β- and ω-oxidation of fatty acids. These findings suggest that STAT6 may functionally antagonize PPAR-α–dependent metabolic processes [90].

This mechanism may be particularly relevant in the context of the skin, where PPAR-α plays a key role in regulating epidermal lipid homeostasis. It regulates keratinocyte differentiation, lipid synthesis, and lamellar body formation, which are essential for lipid transport into the extracellular space of the stratum corneum. Activation of PPAR-α promotes the production of barrier lipids and accelerates epidermal barrier repair.

Importantly, PPAR-α expression is reduced in the skin of patients with AD, particularly within inflammatory lesions, where it may decrease by up to 50% compared to healthy individuals. In addition, PPAR-α contributes to the maintenance of skin barrier function by reducing transepidermal water loss and regulating skin pH. Thus, decreased PPAR-α expression may contribute to the development and persistence of skin lesions in AD through disruption of epidermal lipid pathways [91].

In addition to PPAR-α, the transcription factor sterol regulatory element-binding protein 1 (SREBP-1) is a key regulator of epidermal lipid homeostasis, controlling the expression of enzymes involved in cholesterol and fatty acid synthesis. The SREBP-1c isoform is of particular importance, as it is a direct target of liver X receptors (LXRs), which are expressed in keratinocytes. Activation of LXRs has been shown to enhance epidermal lipid synthesis, promote lamellar body secretion, and facilitate lipid processing within the stratum corneum, all of which are essential for proper barrier function [92]. Impaired activity of lipid-regulating nuclear receptors, including PPAR-α, LXR, and SREBP-1, may significantly contribute to epidermal barrier dysfunction in AD [90,91,92].

#### 4.2.3. Role of ABCA12 in Epidermal Lipid Transport and Lamellar Body Secretion

Efficient lipid utilization in the epidermis depends on its transport into lamellar bodies and subsequent secretion into the intercellular space. ATP-binding cassette transporter A12 (ABCA12), a member of the ATP-binding cassette (ABC) transporter family, plays a critical role in this process by mediating the transfer of glucosylceramides into lamellar bodies within keratinocytes. Following secretion into the intercellular space of the stratum corneum, these lipids form essential components of the extracellular lipid lamellae that maintain skin barrier integrity.

ABCA12 is expressed throughout most layers of the epidermis. Mutations in ABCA12 underlie severe autosomal recessive disorders such as ichthyosis, characterized by profound lipid barrier defects resulting from impaired lipid transport via lamellar bodies. Experimental models further demonstrate that ABCA12 deficiency leads to intracellular lipid accumulation in keratinocytes and severe disruption of epidermal barrier organization [85].

The regulation of ABCA12 expression in keratinocytes remains not fully understood; however, its expression increases during epidermal differentiation. Activation of nuclear receptors involved in lipid metabolism, including PPARs and LXRs, has been shown to upregulate ABCA12, thereby promoting lamellar body formation and secretion, as well as maintaining epidermal lipid barrier homeostasis [86].

In atopic dermatitis, decreased activity of lipid metabolic pathways is accompanied by reduced PPAR-α expression. This suggests that impaired nuclear receptor signaling may indirectly contribute to reduced ABCA12 function and compromised lipid transport in the epidermis.

Therefore, proper ABCA12-dependent lipid transport is essential for the assembly and secretion of epidermal barrier lipids. In AD, dysregulation of pathways controlling ABCA12 may further impair lamellar body function, contributing to defective extracellular lipid organization and barrier dysfunction [85,86].

### 4.3. Omics Evidence of Lipid Dysregulation in Atopic Dermatitis

Lipidomic, metabolomic, and transcriptomic studies collectively provide converging evidence that alterations in ceramide composition, fatty acid elongation, and barrier-related gene expression in atopic dermatitis are associated with Th2-driven inflammatory pathways [93].

#### 4.3.1. Lipidomic Insights

Lipidomic studies provide evidence that IL-4/IL-13 signaling is associated with significant alterations in epidermal lipid composition in atopic dermatitis. These include shifts in fatty acid chain length and reductions in key lipid classes, such as ceramides, sphingomyelin, and lysophosphatidylcholine. Notably, these abnormalities are observed in both lesional and non-lesional skin, suggesting that lipid dysregulation reflects a systemic feature of the disease rather than a purely localized phenomenon [17].

Another study by Emmert et al. [15] highlighted spatial variability in epidermal lipid composition across different anatomical sites in AD. The analysis revealed changes in lipid profiles, including increased levels of total ceramides, free fatty acids, and cholesterol sulfate. However, these findings should be interpreted with caution, as most studies report an overall decrease in ceramide levels, primarily due to reduced long-chain species. The absence of long-chain ceramides in the analytical panel likely explains this discrepancy.

Furthermore, ceramide composition varies depending on the sampling site, and AD skin exhibits distinct patterns of lipid dysregulation compared to healthy controls, indicating that these alterations are region-specific and influenced by local skin characteristics. Overall, lipid abnormalities in AD are compositionally complex and vary across anatomical sites [15].

#### 4.3.2. Transcriptomic Insights

Building on lipidomic findings, transcriptomic studies further elucidate molecular alterations in AD by revealing widespread changes in gene expression in the skin. Cole et al. identified tissue-specific transcriptional alterations, with numerous differentially expressed genes primarily related to immune responses and extracellular processes, while genes involved in lipid metabolism were relatively underrepresented [94,95].

Another transcriptomic study by Tsoi et al. [49] showed that AD is characterized by substantial transcriptomic heterogeneity, with gene expression profiles varying according to disease stage and lesion characteristics. Principal component analysis revealed that AD skin lesions exhibit greater transcriptomic heterogeneity than psoriasis, along with a higher proportion of dysregulated long non-coding RNAs (lncRNAs). Notably, a significant overlap in transcriptomic signatures was observed between AD and psoriasis, particularly in genes associated with inflammatory responses and epidermal function, suggesting shared molecular mechanisms despite distinct clinical phenotypes.

Taken together, these findings indicate coordinated alterations in gene expression and lipid metabolism in AD, supporting a role for Th2-driven immune responses in shaping epidermal function.

#### 4.3.3. Metabolomic Insights

Metabolomic studies complement lipidomic and transcriptomic analyses by providing a comprehensive view of alterations in metabolic pathways. In atopic diseases, including AD, widespread disturbances in metabolites and associated biochemical processes have been reported [95].

In AD, systemic metabolic alterations associated with inflammation and lipid metabolism have been observed, including increased levels of eicosanoids such as leukotriene B4 and prostaglandins, as well as conjugated bile acids. These changes may reflect enhanced inflammatory activity and metabolic dysregulation. Furthermore, differences in metabolite profiles according to IgE levels suggest heterogeneity between disease endotypes [96].

However, these systemic alterations appear to influence epidermal barrier function indirectly, rather than representing a direct mechanistic link, as they primarily reflect circulating inflammatory mediators and metabolic changes rather than local dysregulation of lipid synthesis and organization within keratinocytes.

#### 4.3.4. Integrative Perspective and Heterogeneity

While these alterations are not observed uniformly across all patients, the overall trend supports a model of cytokine-driven changes in epidermal metabolism. It should be emphasized that alterations in lipid composition, protein expression, metabolism, and transcriptomic profiles are heterogeneous and vary between individuals with AD, reflecting the considerable clinical and biological diversity of the disease.

Nevertheless, converging evidence indicates that increased IL-4/IL-13 signaling is associated with coordinated metabolic and structural changes affecting the epidermal barrier. These findings support the concept of inflammation-associated metabolic changes as a dynamic process rather than a purely static structural defect [17,97,98].

Importantly, the lipid abnormalities observed in AD are likely multifactorial and cannot be attributed exclusively to IL-4/IL-13 signaling. In addition to Th2 cytokine-mediated effects, epidermal lipid alterations may also be influenced by filaggrin deficiency, impaired keratinocyte differentiation, altered skin pH, microbial colonization, mechanical damage caused by scratching, previous topical treatment, disease severity, and anatomical site of sample collection [99,100]. Therefore, although omics-based and experimental studies support an important association between Th2-driven inflammation and epidermal lipid dysregulation, these findings should be interpreted within the broader inflammatory and barrier-disrupted microenvironment characteristics of AD [14,101].

The major experimental and omics-based evidence linking IL-4/IL-13 signaling with epidermal lipid dysregulation in AD is summarized in Table 3.

## 5. Structural and Functional Consequences of Epidermal Lipid Dysregulation

### 5.1. Lipid-Driven Barrier Dysfunction and Clinical Manifestations

Disturbances in epidermal lipid composition in atopic dermatitis, particularly the reduced proportion of hydrophobic ceramides containing very-long-chain fatty acids, lead to disorganization of the lamellar structure of the stratum corneum. These alterations contribute to barrier impairment and are closely linked to the development and persistence of inflammation in AD [17,102,103].

These structural abnormalities translate into key clinical features of AD, including xerosis, pruritus, and alterations in the pH of the stratum corneum. Increased skin permeability facilitates transepidermal water loss and the penetration of irritants and allergens, further promoting inflammatory responses and symptom exacerbation [104,105].

Pruritus is a central feature of AD and is associated with a lowered itch threshold, with scratching triggered by minimal stimuli such as irritants, humidity changes, or sweating. Mechanical injury resulting from scratching further activates keratinocytes and promotes the release of proinflammatory mediators, thereby exacerbating barrier dysfunction and inflammation [3,7,89,104,105]. In addition to barrier disruption, pruritus in atopic dermatitis is strongly associated with neuroimmune interactions involving Th2 cytokines, sensory nerve activation, and epithelial-derived mediators. Among these, IL-31 is considered a key pruritogenic cytokine, as it directly activates sensory neurons and contributes to chronic itch. IL-4 and IL-13 may further enhance neuronal sensitization and amplify its signaling through modulation of sensory pathways [106,107]. Keratinocyte-derived cytokines, including thymic stromal lymphopoietin (TSLP), also contribute to communication between the epidermal barrier and peripheral sensory nerves. Therefore, pruritus in AD is currently regarded as a multifactorial process involving epidermal barrier dysfunction, immune activation, microbial dysbiosis, and neuroimmune signaling, all of which contribute to the self-perpetuating itch–scratch cycle and disease chronicity [24,108].

Alterations in stratum corneum pH may contribute to barrier dysfunction [89]. In AD, increased skin pH enhances the activity of serine proteases, particularly kallikreins, leading to degradation of structural proteins and lipid-processing enzymes, which further compromises epidermal integrity [102].

In addition, reduced levels of antimicrobial peptides in AD promote microbial dysbiosis, particularly colonization by *Staphylococcus aureus*, which is observed in more than 90% of patients [102,104]. The presence of *S. aureus* exacerbates inflammation and further impairs epidermal integrity through multiple mechanisms, including protease activity and modulation of host immune responses. Moreover, *S. aureus* colonization contributes to pruritus by increasing IL-31 expression, thereby reinforcing inflammatory and itch responses in AD. These effects are closely linked to lipid abnormalities in AD, as reduced levels of antimicrobial lipids facilitate bacterial colonization and amplify inflammation. These interactions are discussed in more detail in the following section [109].

### 5.2. Lipid-Microbiome Interactions and Inflammatory Feedback

Secondary epidermal barrier dysfunction associated with disturbances in lipid composition may influence the survival and composition of microorganisms in the stratum corneum [29,110].

In addition to pathogenic bacteria, commensal microorganisms such as *Staphylococcus epidermidis* may also contribute to epidermal barrier homeostasis through interactions with host lipid metabolism. Experimental studies demonstrated that *S. epidermidis* secretes sphingomyelinase capable of promoting the conversion of sphingomyelin into protective ceramides, thereby supporting stratum corneum hydration and barrier integrity [111].

Sphingosine is a key lipid with antibacterial properties that plays an important role in the innate immune defense of the epidermis, highlighting that epidermal lipids not only contribute to structural barrier integrity but also directly regulate microbial colonization and host-microbe interactions [87,112]. In atopic dermatitis, the skin exhibits a markedly disturbed microbial balance characterized by reduced diversity, including decreased abundance of genera such as *Cutibacterium*, *Streptococcus*, *Acinetobacter*, *Corynebacterium*, and *Prevotella*, alongside an increased prevalence of *Staphylococcus*, particularly *S. aureus* [113,114].

In humans, the abundance of *Staphylococcus aureus* on the skin is inversely correlated with levels of antimicrobial lipids, such as sphingosine and sapienic acid. Notably, reduced concentrations of these lipids are observed in individuals with AD and are associated with increased susceptibility to *S. aureus* colonization [87,88].

*Staphylococcus aureus* disrupts host immune responses and directly impairs epidermal barrier integrity through multiple mechanisms. These include the production of α-toxin, which induces IL-1R-dependent inflammation and limits the accumulation of *S. aureus*-specific regulatory T cells (Tregs), as well as the secretion of proteases, including serine proteases and kallikreins, which promote degradation of the stratum corneum and facilitate barrier penetration [113,115,116].

In addition, lipoteichoic acid (LTA) produced by the bacterial cell wall inhibits the expression of epidermal barrier proteins, further contributing to barrier dysfunction.

Moreover, *S. aureus* isolated from patients with AD exhibits a distinct surface protein profile, with proinflammatory lipoproteins that induce TSLP expression. This, in turn, modulates immune responses via Langerhans cells and contributes to a Th1/Th2 imbalance [115,116].

Th2 cytokines, including IL-4, increase the expression of fibronectin and fibrinogen in the skin of patients with AD, thereby enhancing the adhesion of *Staphylococcus aureus* to the stratum corneum. Consequently, *S. aureus* colonization is both facilitated by barrier dysfunction and further contributes to its progression through enzyme-mediated damage, increased bacterial adhesion, and dysregulation of host immune responses. Importantly, these processes are closely linked to lipid abnormalities in AD, as reduced levels of antimicrobial lipids facilitate bacterial colonization and amplify inflammation [116].

Keratinocytes act as key amplifiers of type 2 inflammation. In this context, bacterial dysbiosis in AD contributes to barrier dysfunction and increased exposure of the skin immune system to microorganisms and allergens, thereby promoting immune activation. Keratinocyte damage and activation, partly induced by *Staphylococcus aureus*-derived factors, lead to the production of proinflammatory cytokines and epithelial alarmins, as described in Section 3.3, further amplifying immune activation.

As a result, resident dendritic cells become activated and promote the differentiation of naive T lymphocytes toward a Th2/Th22-type inflammatory response. The resulting production of type 2 cytokines, including IL-4, IL-5, and IL-13, drives further recruitment of immune cells such as eosinophils, mast cells, and B lymphocytes, amplifying the inflammatory cascade. This inflammatory state further impairs epidermal barrier function and promotes colonization by pathogenic bacteria, particularly *Staphylococcus aureus*, which in turn enhances keratinocyte activation and perpetuates inflammation.

**Figure 3:**
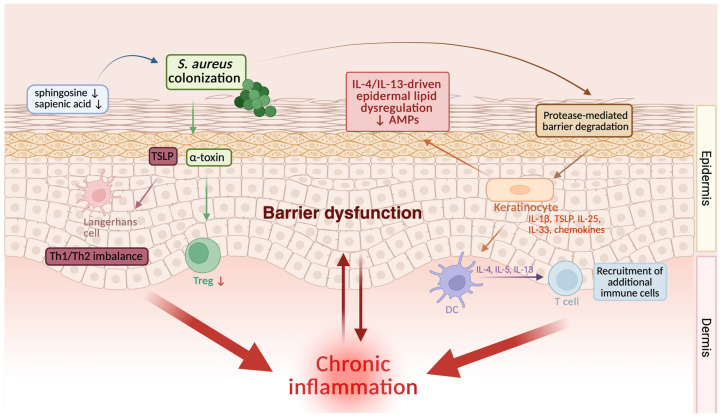
Pathogenic Cycle of Type 2 Inflammation, Epidermal Lipid Dysregulation, and Microbial Dysbiosis in Atopic Dermatitis. Created in BioRender. Andrzejczak, K. (2026) https://BioRender.com/p6qe2dn. Abbreviations: AMPs, antimicrobial peptides; DC, dendritic cell; IL, interleukin; *S. aureus*, *Staphylococcus aureus*; Th, T helper; Treg, regulatory T cell; TSLP, thymic stromal lymphopoietin.

Together, these observations indicate that inflammation-driven alterations in epidermal lipid composition actively shape the skin microbiome and contribute to the establishment of a self-reinforcing pathogenic cycle linking lipid dysregulation, microbial imbalance, and chronic inflammation in AD, as illustrated in Figure 3 [101,113,114].

## 6. Therapeutic Implications and Future Perspectives

### 6.1. Restoring Barrier Function

Therapeutic strategies aimed at supporting epidermal lipid homeostasis may contribute to improved skin barrier function and complement anti-inflammatory treatment in atopic dermatitis, although their independent impact on disease control requires further validation. Disturbances in stratum corneum lipid organization, largely driven by type 2 cytokines, lead to impaired epidermal barrier function. Therefore, effective management may require simultaneous targeting of both inflammation and lipid metabolism in keratinocytes. Targeted inhibition of IL-4/IL-13 signaling and broader modulation of cytokine-dependent JAK/STAT pathways have both been associated with improvement in barrier function and partial restoration of epidermal homeostasis. This highlights the role of epidermal lipids as an important link between inflammation and barrier dysfunction, supporting further investigation of lipid-related pathways as potential therapeutic targets [43,46,117,118,119,120].

#### 6.1.1. Topical Moisturizers as Barrier Support Therapies

Treatment of atopic dermatitis involves controlling inflammation, avoiding triggers, and supporting epidermal barrier function, in which topical moisturizers, such as emollients, occlusive agents, and humectants, play a crucial role. These products reduce TEWL, improve epidermal hydration, and partially replenish intercellular lipids, resulting in symptom relief, prolongation of time to flare-up, and reduced need for topical glucocorticosteroids. However, they are most effective when combined with anti-inflammatory therapy and remain insufficient as monotherapy [1,121,122,123,124].

Despite their widespread clinical use, the ability of emollients to permanently restore the epidermal barrier remains limited. Their impact on the composition and organization of stratum corneum lipids is variable, as demonstrated by multiple analyses, including a Cochrane meta-analysis, highlighting substantial heterogeneity in outcomes [123].

Lipid-rich formulations may partially support barrier repair, but their effects are primarily compensatory. Occlusive emollients reduce water loss by forming a hydrophobic film on the skin surface, whereas formulations containing physiological lipids show greater potential to modulate the structure of the lipid matrix of the stratum corneum [119,120,125,126,127].

Clinical and lipidomic studies indicate that these preparations may improve the integrity of the epidermal barrier and partially normalize the lipid profile of the stratum corneum, which correlates with reduced TEWL and sensitivity to irritants. However, their effectiveness depends on the relative proportions of cholesterol, ceramides, and free fatty acids and does not lead to complete reactivation of endogenous lipid synthesis or sustained restoration of barrier function [117,128].

Because type 2 cytokines disrupt epidermal lipid biosynthesis at the cellular level, emollients provide primarily symptomatic relief and do not directly target the underlying pathogenic mechanisms of AD. Therefore, they should be considered adjunctive therapy and used synergistically with anti-inflammatory treatment [14,17,123,129].

#### 6.1.2. Targeting the IL-4/IL-13 Axis

IL-4- and IL-13-targeted therapies play a key role in atopic dermatitis by targeting the central Th2-dependent pathway. Chronic activation of this axis sustains inflammation and impairs epidermal barrier function, including keratinocyte differentiation and stratum corneum lipid organization. Thus, blocking IL-4/IL-13 signaling targets the mechanism linking inflammation with barrier dysfunction [12,14,98,130,131].

Biological therapies targeting the Th2 pathway include dupilumab, lebrikizumab, and tralokinumab, whose efficacy has been demonstrated in numerous randomized controlled trials (RCTs) and real-world studies, with the most extensive real-world evidence (RWE) available for dupilumab [51,132,133,134,135,136,137,138,139,140].

Dupilumab, a monoclonal antibody targeting the IL-4Rα subunit, blocks both IL-4 and IL-13 signaling, whereas tralokinumab and lebrikizumab neutralize IL-13 by preventing signaling through the IL-4Rα/IL-13Rα1 receptor complex. However, they differ in their IL-13 binding profiles: tralokinumab blocks IL-13 interaction with both IL-13Rα1 and IL-13Rα2, whereas lebrikizumab selectively inhibits the IL-4Rα/IL-13Rα1 signaling complex without affecting IL-13 binding to IL-13Rα2. As a result, these agents limit Th2 axis activation and modulate mechanisms underlying epidermal barrier dysfunction [133,136,141,142,143,144].

Evidence regarding barrier-related effects of selective IL-13 blockade is also emerging. In patients with moderate-to-severe AD, tralokinumab treatment has been associated with improvement in skin barrier function, including reduced transepidermal water loss, increased stratum corneum hydration, reductions in selected barrier dysfunction-associated and proinflammatory biomarkers, and reduced *Staphylococcus aureus* abundance [145]. By contrast, currently available translational data for lebrikizumab more clearly document systemic biomarker modulation than direct barrier lipid recovery. In patients with moderate-to-severe AD, selective IL-13 inhibition with lebrikizumab induced progressive reductions in circulating type 2 inflammatory biomarkers, including CCL13, CCL17, CCL22, and periostin, which correlated with clinical improvement. Reductions in CCL26 were also correlated with clinical improvement, and CCL26 was identified as a pharmacodynamic marker of lebrikizumab response. However, direct evidence regarding epidermal lipid normalization or barrier lipid remodeling remains limited [146].

More recent therapeutic strategies include eblasakimab, a monoclonal antibody targeting IL-13Rα1, which, unlike ligand-neutralizing antibodies, directly inhibits signaling through the type II receptor complex, thereby blocking signaling mediated by both IL-13 and IL-4 [147,148,149].

Clinical studies have shown that blocking the IL-4/IL-13 axis with dupilumab leads to significant improvements in epidermal barrier function, including a reduction in TEWL to levels comparable to those observed in healthy individuals, increased stratum corneum hydration, and improvements in lipid composition, including elongation of fatty acid chains and partial normalization of the ceramide profile. These changes have been associated with improvement in very-long-chain fatty acid biosynthesis, likely through relief of cytokine-mediated inhibition of elongases (ELOVL3 and ELOVL6). These effects have been observed in both lesional and non-lesional skin, and improvements in lipid profile correlate with reductions in TEWL, highlighting the close relationship between IL-4/IL-13 signaling, epidermal lipid metabolism, and skin barrier function [98,150,151].

In addition to clinical observations, emerging omics data provide insight into the molecular effects of IL-4/IL-13 blockade in atopic dermatitis. These studies indicate that metabolic alterations in AD are dynamically modulated by Th2-targeted therapy and may be more pronounced in treatment responders. Zhang et al. [152] demonstrated that dupilumab treatment is associated with significant changes in metabolomic and lipidomic profiles that correlate with clinical response, particularly affecting pathways involved in glycerophospholipid, sphingolipid, and arachidonic acid metabolism. These changes likely reflect reduced cutaneous inflammation.

IL-4/IL-13 blockade induces profound molecular remodeling of the skin. Longitudinal analyses by Goleva et al. demonstrated that dupilumab therapy restores the expression of proteins involved in epidermal barrier formation, lysosomal enzymes essential for lamellar body formation, and proteins associated with oxidative responses, while reducing markers of epidermal hyperplasia and metabolic activation [153].

This is supported by additional reports showing restoration of barrier-related and structural proteins, including those involved in cornified envelope formation and ceramide organization, alongside normalization of enzymes involved in lipid metabolism and lamellar body formation [97].

Moreover, atopic dermatitis (AD) skin is characterized by altered ceramide composition, with a predominance of short-chain species and a reduction in long-chain, highly hydrophobic ceramides. Importantly, these alterations appear to be strongly influenced by type 2 inflammation and may be at least partly reversible during dupilumab therapy, further supporting a role for IL-4/IL-13 blockade in modulating lipid metabolism and epidermal barrier homeostasis [97].

Although these observations support a close relationship between type 2 cytokine blockade, barrier recovery, and normalization of epidermal lipid profiles, the underlying directionality remains incompletely resolved. Lipid improvement may reflect, at least in part, direct relief of IL-4/IL-13-mediated suppression of keratinocyte lipid metabolic pathways; however, it may also arise secondarily from broader reduction in cutaneous inflammation and restoration of epidermal homeostasis. Available therapeutic lipidomic studies remain limited by relatively small cohorts, short follow-up periods, and a strong predominance of dupilumab-based datasets, with comparatively sparse data for other therapies targeting the IL-4/IL-13 axis [17,97,98,152].

Taken together, current findings suggest that therapies targeting the IL-4/IL-13 axis may support epidermal barrier restoration through mechanisms that could extend beyond inflammation control alone. Further studies are needed to define their long-term impact on epidermal lipid metabolism and barrier function, including comparative analyses of individual agents and their mechanisms of action [53,98,131].

#### 6.1.3. JAK/STAT Inhibition and Lipid Metabolism

As previously discussed, type 2 cytokine signaling via the JAK/STAT pathway plays an important role in regulating inflammation and lipid metabolism in keratinocytes. In this context, JAK inhibitors represent a novel therapeutic class in atopic dermatitis. They can be divided into first-generation agents, which act non-selectively on multiple JAK family kinases, and newer, more selective inhibitors targeting specific kinases [60,154,155,156]. By inhibiting intracellular signaling pathways of multiple cytokines, these agents enable broad modulation of inflammatory responses and can be administered both systemically and topically [157].

Importantly, the effects of JAK inhibitors on epidermal barrier recovery and lipid-related pathways should not be interpreted solely as a consequence of IL-4/IL-13 signal suppression. Unlike cytokine-specific biologics, JAK inhibitors modulate intracellular signaling downstream of multiple inflammatory mediators relevant to AD, including IL-4, IL-13, IL-31, IL-22, interferon-related signaling, and other cytokine networks. Therefore, any improvement in epidermal lipid metabolism observed during JAK inhibition may reflect broader inflammatory reprogramming rather than a selective reversal of IL-4/IL-13-driven lipid dysregulation [62].

Approved systemic therapies include abrocitinib and upadacitinib (selective JAK1 inhibitors) and baricitinib (JAK1/2). Their efficacy has been demonstrated in clinical trials [158,159,160,161,162,163,164,165].

Unlike biologics that act extracellularly, JAK inhibitors penetrate cells and modulate intracellular signaling, enabling rapid effects on gene expression related to epidermal barrier function. Inhibition of JAK kinase activity reduces STAT phosphorylation and promotes restoration of gene expression involved in terminal epidermal differentiation [166,167]. Clinical observations indicate that this process correlates with reduced TEWL and improvements in clinical outcomes [168,169].

Tofacitinib is a first-generation, broad-spectrum JAK inhibitor showing the highest affinity for JAK1 and JAK3 and lower affinity for JAK2 and TYK2. Clinical studies have shown that it may be effective in moderate-to-severe atopic dermatitis when administered orally, and in milder forms when used topically. However, its use in AD is based on a limited number of studies, and large randomized controlled trials evaluating its efficacy and safety are lacking [62,156,157,170].

Experimental data provide further insight into its effects on lipid metabolism. A study by Flori et al. [60] demonstrated that stimulation of keratinocytes with Th2 cytokines led to activation of STAT3 and STAT6 and altered expression of genes associated with lipid metabolism, including a significant reduction in expression of fatty acid elongases (ELOVL1, ELOVL3, and ELOVL4). Ceramide profiles, however, showed only minor changes. Tofacitinib reduced STAT activation and partially reversed these effects, restoring the expression of enzymes involved in lipid synthesis. These findings indicate that inhibition of the JAK-STAT pathway may modulate Th2 cytokine-induced dysregulation of lipid metabolism and support epidermal barrier function.

However, the available data are limited and derive primarily from experimental studies, underscoring the need for further clinical research to evaluate the relevance of these findings in patients with AD [14,17,60].

At present, available evidence does not allow direct comparison of the extent, timing, anatomical distribution, lipid subtype specificity, or long-term durability of epidermal lipid restoration across dupilumab, tralokinumab, lebrikizumab, eblasakimab, and JAK inhibitors. Among these therapies, dupilumab currently provides the most developed clinical and translational evidence linking treatment to improvement in epidermal barrier parameters and lipid-related profiles. For tralokinumab, available human data support improvement in barrier physiology and selected stratum corneum biomarkers, whereas currently available data for lebrikizumab more clearly document modulation of inflammatory biomarkers than direct epidermal lipid remodeling. For eblasakimab, evidence regarding epidermal lipid restoration remains insufficiently defined. In contrast, lipid-related effects of JAK inhibition remain supported mainly by experimental evidence, particularly in Th2-stimulated epidermal models treated with tofacitinib. Overall, available data support a beneficial effect of IL-4/IL-13-targeted therapies on skin barrier function, whereas evidence for JAK inhibitors remains more limited and is not yet sufficient to define their role in barrier restoration conclusively. Dedicated comparative studies are therefore needed to determine whether these therapeutic classes differ in the degree, kinetics, anatomical site, lipid-subclass specificity, or durability of epidermal lipid restoration [60,93,98,131,145,146,147,149,152,171].

### 6.2. Integrated Therapy Approach

Given the close interplay between inflammation and epidermal barrier dysfunction in AD, therapeutic strategies may be most effective when anti-inflammatory treatment is combined with barrier-directed support. By reducing cytokine-mediated impairment of keratinocyte function, anti-inflammatory therapies may facilitate partial recovery of endogenous lipid and barrier protein synthesis, thereby creating a more favorable context for epidermal barrier repair.

Exogenous administration of physiological lipids (ceramides, cholesterol, and free fatty acids) may support repair of the lipid matrix of the stratum corneum and partially compensate for their deficiency. Combination therapy may also reduce treatment-related adverse effects, including epidermal atrophy associated with topical corticosteroids.

Furthermore, this approach may favorably influence the skin microbiome by limiting colonization by *Staphylococcus aureus* and promoting restoration of the commensal microbiota. Studies indicate that it is associated with improved disease control and a steroid-sparing effect.

**Figure 4:**
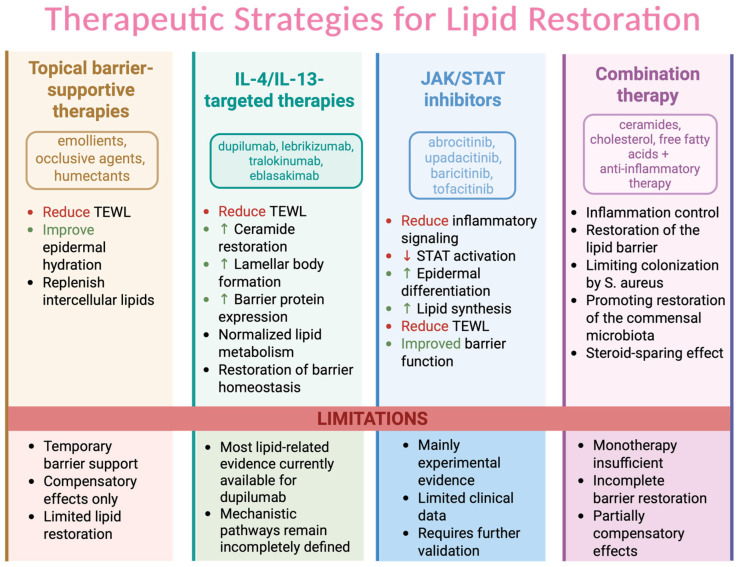
Therapeutic strategies supporting epidermal lipid restoration and barrier repair in atopic dermatitis. Created in BioRender. Andrzejczak, K. (2026) https://BioRender.com/yaicugp. Abbreviations: IL, interleukin; JAK, Janus kinase; STAT, signal transducer and activator of transcription; *S. aureus*, *Staphylococcus aureus*; TEWL, transepidermal water loss.

In summary, effective treatment of atopic dermatitis may benefit from a two-pronged approach combining inflammation control with support of epidermal lipid barrier repair. Disturbances in epidermal lipid metabolism represent a key link between type 2 immune responses and barrier dysfunction, highlighting the importance of therapies targeting not only inflammation but also keratinocyte function as well as epidermal lipid metabolism. The therapeutic strategies discussed above and their potential contributions to epidermal lipid restoration and barrier repair are summarized in Figure 4 [14,17,21,77,129,172,173,174,175,176,177].

### 6.3. Future Directions: Lipidomic Profiles as Potential Biomarkers for Patient Stratification

Traditional approaches to the management of diseases associated with epidermal barrier dysfunction have relied primarily on clinical phenotyping and empirical therapy selection. Advances in high-throughput analytical techniques, particularly liquid chromatography-tandem mass spectrometry (LC-MS/MS), have enabled the development of stratum corneum lipidomics, allowing quantitative and qualitative analyses of lipid classes, including ceramides, free fatty acids, and cholesterol. These advances provide an important research framework for exploring lipid profiles as candidate biomarkers for patient stratification and for supporting future precision medicine approaches; however, their clinical applicability remains to be established [178,179,180].

Lipidomic studies indicate significant heterogeneity in epidermal barrier dysfunction, associated with disruption of lamellar organization and changes in physicochemical properties. These alterations are partially regulated by inflammatory pathways, particularly type 2 immune pathways, as well as by local and systemic inflammatory processes [15,17,120,178].

Quantitative and qualitative changes in ceramides play a significant role in epidermal barrier dysfunction and are closely linked to barrier function, including skin hydration and TEWL. In this context, ceramide alterations may represent promising candidate biomarkers for patient stratification and recurrence risk assessment, although their predictive value requires validation in larger, standardized, longitudinal studies [14,181].

A promising research direction is the integration of lipidomics with other molecular data to identify candidate biomarkers that may be associated with disease severity, inflammatory endotype, and treatment response. Multimodal approaches combining lipidomic data with transcriptomic, proteomic, and immunological profiles may provide a more comprehensive characterization of AD heterogeneity, particularly when using minimally invasive techniques such as tape stripping.

Despite its potential translational relevance, the application of lipidomics in clinical practice remains limited by the lack of methodological standardization in sampling and analytical workflows, site-to-site variability in epidermal lipid composition, high costs and limited accessibility of specialized platforms, incomplete reproducibility across analytical methods, and the absence of validated clinically applicable thresholds, all of which currently hinder biomarker validation and routine implementation [15,98,182,183].

In the future, integration of molecular data may support the development of more targeted therapeutic strategies, provided that lipidomic signatures are validated across standardized and clinically relevant cohorts [180,184].

## 7. Conclusions

Atopic dermatitis is a complex inflammatory skin disease in which immune dysregulation and epidermal barrier impairment are closely interconnected. Accumulating evidence indicates that IL-4/IL-13 signaling, particularly through the JAK-STAT6 pathway, plays an important role in keratinocyte dysfunction, including disturbances in lipid metabolism and epidermal differentiation.

These cytokine-driven effects appear to extend beyond classical immune activation and may affect key pathways of lipid metabolism, including fatty acid elongation and ceramide synthesis, leading to alterations in the stratum corneum lipid matrix, increased transepidermal water loss, and enhanced susceptibility to microbial colonization and inflammation.

Notably, these findings support the concept that epidermal barrier dysfunction represents a critical component of disease pathogenesis that is functionally linked with immune dysregulation and may also arise as a secondary consequence of cytokine-driven inflammation, rather than being solely a primary structural defect. This integrated perspective emphasizes the need to consider both barrier impairment and immune activation as interdependent processes, moving beyond the traditional “outside-in” and “inside-out” paradigms toward a more dynamic and interconnected model of disease pathogenesis.

However, it is important to emphasize that the mechanistic link between IL-4/IL-13 signaling and epidermal lipid dysregulation, although supported by converging mechanistic, experimental, and clinical evidence, remains incompletely defined. Current data support a strong association but do not fully establish causality, and it remains to be determined to what extent these alterations reflect coordinated metabolic reprogramming versus broader inflammation-induced dysregulation.

Furthermore, the interplay between IL-4/IL-13-dependent lipid dysregulation, microbial imbalance, and activation of the immune response establishes a self-perpetuating cycle that contributes to disease chronicity. Understanding these interactions provides a more comprehensive view of AD pathogenesis and underscores the importance of targeting both inflammation and epidermal lipid metabolism in therapeutic strategies.

Future research should focus on further clarifying cytokine-induced metabolic alterations in keratinocytes and their role in barrier dysfunction. Emerging lipidomic and integrative omics approaches may facilitate the identification of candidate biomarkers and support the future development of more targeted therapeutic strategies, provided that these findings are validated in standardized clinical studies.

## 8. Materials and Methods

This article was designed as a narrative review providing a comprehensive synthesis of current evidence on the interplay between IL-4/IL-13-driven type 2 inflammation, epidermal lipid metabolism, and skin barrier dysfunction in atopic dermatitis. It integrates mechanistic, translational, omics-based, and therapeutic findings relevant to cytokine-mediated alterations in keratinocyte lipid pathways. This narrative approach was selected to enable an integrated, conceptually focused synthesis of evidence from heterogeneous mechanistic, translational, omics-based, and clinical studies.

Relevant publications were identified through searches of major biomedical and multidisciplinary databases, including PubMed, Google Scholar, Web of Science, Embase, and Scopus. The literature search was performed using combinations of the following keywords and Medical Subject Headings (MeSH): “atopic dermatitis”, “AD”, “IL-4”, “IL-13”, “type 2 inflammation”, “Th2 cytokines”, “JAK-STAT signaling”, “STAT6”, “keratinocytes”, “epidermal lipid metabolism”, “skin barrier dysfunction”, “ceramides”, “very-long-chain fatty acids”, “ELOVL”, “ceramide synthase”, “CerS3”, “ABCA12”, “PPAR-α”, “SREBP-1”, “lipidomics”, “metabolomics”, “transcriptomics”, “transepidermal water loss”, “dupilumab”, “tralokinumab”, “lebrikizumab”, “eblasakimab”, “JAK inhibitors”, “upadacitinib”, “abrocitinib”, “baricitinib”, “tofacitinib”, “skin microbiome”, “*Staphylococcus aureus*”, and “*Staphylococcus epidermidis*”.

The review focused primarily on publications from the last decade in order to reflect recent advances in the understanding of AD immunopathogenesis, epidermal lipid biology, and targeted therapeutic strategies. Earlier landmark studies were also included where necessary to provide foundational mechanistic context, particularly in relation to epidermal barrier organization, ceramide biology, and keratinocyte lipid metabolism. Priority was given to original experimental studies, translational analyses of human AD skin, omics-based investigations, and clinical studies evaluating therapies targeting the IL-4/IL-13 axis or JAK-STAT signaling. Review articles were used selectively to provide broader context and to facilitate the identification of relevant primary literature.

Studies were considered particularly relevant if they addressed IL-4/IL-13-mediated signaling in keratinocytes, regulation of epidermal lipid metabolism, ceramide and very-long-chain fatty acid abnormalities, omics-based alterations in AD skin, functional measures of barrier impairment such as transepidermal water loss and stratum corneum hydration, microbiome–lipid interactions, or therapeutic modulation of epidermal lipid pathways and barrier repair by IL-4/IL-13-targeted therapies and JAK/STAT inhibition. Evidence was interpreted according to study type: mechanistic experimental studies were used primarily to support biological plausibility, human omics and translational studies were used to identify disease-associated lipid and molecular patterns, and clinical studies were used to evaluate therapeutic relevance.

As this article was designed as a narrative review, no formal review protocol was registered, and no quantitative meta-analysis or formal risk-of-bias assessment was performed. Study selection was guided by mechanistic relevance, methodological robustness, and contribution to understanding the relationship between IL-4/IL-13 signaling, epidermal lipid metabolism, and epidermal barrier dysfunction in AD. Particular attention was given to distinguishing mechanistic experimental evidence from associative human omics and clinical data, especially when discussing the potential clinical relevance of lipidomic findings and therapy-associated effects on epidermal lipid pathways and barrier function.

Figure 1, Figure 2, Figure 3 and Figure 4 were created using BioRender.com.

## Figures and Tables

**Table 1:** Structural and functional characteristics of EOS and EOP ceramide subclasses in the epidermal barrier.

Ceramide Subclass	Structure	Main Role in the Epidermis	References
EOS	esterified ω-hydroxy fatty acid + sphingosine	Lamellar membrane organization and CLE formation	[79]
EOP	esterified ω-hydroxy fatty acid + phytosphingosine	Epidermal barrier stabilization and CLE organization	[79]

Abbreviations: CLE, corneocyte lipid envelope; EOS, esterified ω-hydroxy acyl-sphingosine; EOP, esterified ω-hydroxy acyl-phytosphingosine.

**Table 2:** Major lipid abnormalities associated with epidermal barrier dysfunction in atopic dermatitis.

Lipid Components	Alteration in AD	Functional Consequences	References
Ceramides (total)	Reduced levels	Impaired barrier integrity and increased TEWL	[77,83,84]
EOS/EOP ceramides	Reduced ω-O-acylceramides and shorter fatty acid chains	Lamellar disorganization and defective CLE formation	[17,22,46]
Very-long-chain fatty acids	Reduced elongation and chain shortening	Altered lipid packing and barrier permeability	[39,83]
Cholesterol and free fatty acids	Altered extracellular lipid organization	Impaired lamellar membrane stability	[21,81]
Sphingomyelin and lysophosphatidylcholine	Reduced levels	Disturbed epidermal lipid composition	[17]
Lamellar body processing/ABCA12	Impaired lipid transport and secretion	Defective extracellular lipid assembly	[85,86]
Antimicrobial lipids	Reduced antimicrobial lipids (e.g., sphingosine, sapienic acid)	Increased microbial colonization and inflammation	[87,88]

Abbreviations: ABCA12, ATP-binding cassette transporter A12; AD, atopic dermatitis; CLE, corneocyte lipid envelope; EOS, esterified ω-hydroxy acyl-sphingosine; EOP, esterified ω-hydroxy acyl-phytosphingosine; TEWL, transepidermal water loss.

**Table 3:** Experimental evidence linking IL-4/IL-13 signaling with epidermal lipid dysregulation in atopic dermatitis.

Evidence Category	Experimental Model/Material		Main Findings	References
Cultured keratinocytes and reconstructed epidermis (in vitro/ex vivo)	Human keratinocytes and reconstructed human epidermis stimulated with IL-4/IL-13		Downregulation of ELOVL3/ELOVL6 and altered lipid metabolism in a STAT6-dependent manner	[17,21]
Animal models	Murine AD-like inflammation models		Altered ceramide composition, shortened lipid chains, disrupted barrier organization, and increased TEWL	[14]
Animal models	CerS3-deficient mice		Loss of ultra-long-chain ceramides and severe barrier dysfunction	[82]
Cellular signaling studies	Primary human keratinocytes and reconstructed human epidermis stimulated with IL-4/IL-13		STAT6 signaling inhibited PPAR-α activity	[17]
Cellular signaling studies	Murine hepatocytes		IL-4/STAT6 signaling inhibited PPAR-α transcriptional activity and fatty acid oxidation pathways	[90]
Experimental epidermal models	Keratinocyte and epidermal experimental models		LXR and PPAR activation promoted lipid synthesis, lamellar body formation, and ABCA12 expression	[92]
Genetic epidermal models	Keratinocyte and genetic ABCA12-deficient epidermal models		ABCA12 deficiency resulted in intracellular lipid accumulation and disrupted epidermal barrier organization	[85]
Omics Evidence				
Omics Type	Experimental model/material	Main Findings	Limitations	References
Lipidomic	Lipidomic analyses of human lesional and non-lesional AD skin	Altered epidermal lipid composition and shortened fatty acid chains	Observational human data; does not establish direct IL-4/IL-13 causality	[17]
Lipidomic	Human lesional and non-lesional skin analyses	Spatial variability in epidermal lipid composition across body site	Lipid profiles varied according to anatomical site and analytical lipid panel	[15]
Transcriptomic	AD skin samples; human transcriptomic analyses	Immune-related transcriptomic changes with fewer lipid metabolism-associated genes	Transcriptomic alterations were heterogeneous between patients and disease stages	[94,95]
Transcriptomic	Lesional AD skin samples	Transcriptomic heterogeneity and overlap with psoriasis-associated pathways	Transcriptomic overlap with psoriasis limits AD-specific interpretation	[49]
Metabolomic	Serum and plasma samples from patients with AD and other atopic diseases	Widespread disturbances in metabolites and associated biochemical pathways	Systemic metabolomic profiles may not directly reflect epidermal lipid metabolism	[95]
Metabolomic	Serum/plasma samples from patients with AD	Increased eicosanoids and altered metabolite profiles associated with IgE levels	Primarily systemic inflammatory markers rather than direct epidermal lipid measurements	[96]

Abbreviations: AD, atopic dermatitis; STAT6, signal transducer and activator of transcription 6; ELOVL, elongation of very long-chain fatty acids protein; CerS3, ceramide synthase 3; ABCA12, ATP-binding cassette transporter A12; TEWL, transepidermal water loss.

## Data Availability

No new data were created or analyzed in this study.

## References

[1] Chu D.K., Schneider L., Asiniwasis R.N., Boguniewicz M., De Benedetto A., Ellison K., Frazier W.T., Greenhawt M., Huynh J., AAAAI/ACAAI JTF Atopic Dermatitis Guideline Panel (2024). Atopic Dermatitis (Eczema) Guidelines: 2023 American Academy of Allergy, Asthma and Immunology/American College of Allergy, Asthma and Immunology Joint Task Force on Practice Parameters GRADE- and Institute of Medicine-Based Recommendations. Ann. Allergy Asthma Immunol..

[2] Bai R., Zheng Y., Dai X. (2025). Atopic Dermatitis: Diagnosis, Molecular Pathogenesis, and Therapeutics. Mol. Biomed..

[3] Langan S.M., Irvine A.D., Weidinger S. (2020). Atopic Dermatitis. Lancet.

[4] Lee H.H., Patel K.R., Singam V., Rastogi S., Silverberg J.I. (2019). A Systematic Review and Meta-Analysis of the Prevalence and Phenotype of Adult-Onset Atopic Dermatitis. J. Am. Acad. Dermatol..

[5] Carr S., Pratt R., White F., Watson W. (2024). Atopic Dermatitis. Allergy Asthma Clin. Immunol..

[6] Kim J., Kim B.E., Leung D.Y.M. (2019). Pathophysiology of Atopic Dermatitis: Clinical Implications. Allergy Asthma Proc..

[7] Weidinger S., Beck L.A., Bieber T., Kabashima K., Irvine A.D. (2018). Atopic Dermatitis. Nat. Rev. Dis. Primers.

[8] Khadka V.D., Key F.M., Romo-González C., Martínez-Gayosso A., Campos-Cabrera B.L., Gerónimo-Gallegos A., Lynn T.C., Durán-McKinster C., Coria-Jiménez R., Lieberman T.D. (2021). The Skin Microbiome of Patients with Atopic Dermatitis Normalizes Gradually During Treatment. Front. Cell. Infect. Microbiol..

[9] Bieber T. (2008). Atopic Dermatitis. N. Engl. J. Med..

[10] Edslev S.M., Agner T., Andersen P.S. (2020). Skin Microbiome in Atopic Dermatitis. Acta Derm. Venereol..

[11] Stefanovic N., Irvine A.D. (2024). Filaggrin and beyond: New Insights into the Skin Barrier in Atopic Dermatitis and Allergic Diseases, from Genetics to Therapeutic Perspectives. Ann. Allergy Asthma Immunol..

[12] Facheris P., Jeffery J., Del Duca E., Guttman-Yassky E. (2023). The Translational Revolution in Atopic Dermatitis: The Paradigm Shift from Pathogenesis to Treatment. Cell. Mol. Immunol..

[13] Khatib C.M., Klein-Petersen A.W., Rønnstad A.T.M., Egeberg A., Christensen M.O., Silverberg J.I., Thomsen S.F., Irvine A.D., Thyssen J.P. (2024). Increased Loss-of-Function Filaggrin Gene Mutation Prevalence in Atopic Dermatitis Patients across Northern Latitudes Indicates Genetic Fitness: A Systematic Review and Meta-Analysis. Exp. Dermatol..

[14] Upadhyay P.R., Seminario-Vidal L., Abe B., Ghobadi C., Sims J.T. (2023). Cytokines and Epidermal Lipid Abnormalities in Atopic Dermatitis: A Systematic Review. Cells.

[15] Emmert H., Baurecht H., Thielking F., Stölzl D., Rodriguez E., Harder I., Proksch E., Weidinger S. (2021). Stratum Corneum Lipidomics Analysis Reveals Altered Ceramide Profile in Atopic Dermatitis Patients across Body Sites with Correlated Changes in Skin Microbiome. Exp. Dermatol..

[16] Menzel M., Mraz V., Vaher H., Geisler C., Menné Bonefeld C. (2024). Metabolic Re-Programming of Keratinocytes in Response to Contact Allergens. Contact Dermat..

[17] Berdyshev E., Goleva E., Bronova I., Dyjack N., Rios C., Jung J., Taylor P., Jeong M., Hall C.F., Richers B.N. (2018). Lipid Abnormalities in Atopic Skin Are Driven by Type 2 Cytokines. JCI Insight.

[18] Cibrian D., de la Fuente H., Sánchez-Madrid F. (2020). Metabolic Pathways That Control Skin Homeostasis and Inflammation. Trends Mol. Med..

[19] Klimitz F.J., Shen Y., Repetto F., Brown S., Knoedler L., Ko C.J., Abu Hussein N., Crisler W.J., Adams T., Kaminski N. (2025). Keratinocytes as Active Regulators of Cutaneous and Mucosal Immunity: A Systematic Review across Inflammatory Epithelial Disorders. Front. Immunol..

[20] Ye J., Lai Y. (2025). Keratinocytes: New Perspectives in Inflammatory Skin Diseases. Trends Mol. Med..

[21] Berdyshev E. (2024). Skin Lipid Barrier: Structure, Function and Metabolism. Allergy Asthma Immunol. Res..

[22] Bouwstra J.A., Nădăban A., Bras W., McCabe C., Bunge A., Gooris G.S. (2023). The Skin Barrier: An Extraordinary Interface with an Exceptional Lipid Organization. Prog. Lipid Res..

[23] Fukuda K., Ito Y., Furuichi Y., Matsui T., Horikawa H., Miyano T., Okada T., van Logtestijn M., Tanaka R.J., Miyawaki A. (2024). Three Stepwise PH Progressions in Stratum Corneum for Homeostatic Maintenance of the Skin. Nat. Commun..

[24] Fujii M. (2020). Current Understanding of Pathophysiological Mechanisms of Atopic Dermatitis: Interactions among Skin Barrier Dysfunction, Immune Abnormalities and Pruritus. Biol. Pharm. Bull..

[25] Beck L.A., Cork M.J., Amagai M., De Benedetto A., Kabashima K., Hamilton J.D., Rossi A.B. (2022). Type 2 Inflammation Contributes to Skin Barrier Dysfunction in Atopic Dermatitis. JID Innov..

[26] Criado P.R., Miot H.A., Bueno-Filho R., Ianhez M., Criado R.F.J., de Castro C.C.S. (2024). Update on the Pathogenesis of Atopic Dermatitis. An. Bras. Dermatol..

[27] van Smeden J., Bouwstra J.A. (2016). Stratum Corneum Lipids: Their Role for the Skin Barrier Function in Healthy Subjects and Atopic Dermatitis Patients. Skin Barrier Function.

[28] Pappas A. (2009). Epidermal Surface Lipids. Derm.-Endocrinol..

[29] Siqueira R.A.G.B., Hradkova I., Leite-Silva V.R., Andréo-Filho N., Lopes P.S. (2025). Skin Lipids and Their Influence on Skin Microbiome and Skin Care. ACS Omega.

[30] Feingold K.R., Elias P.M. (2024). The Role of Ceramides in the Disruption of the Cutaneous Permeability Barrier, a Common Manifestation of Skin Disorders. J. Lipid Res..

[31] Feingold K.R. (2012). Lamellar Bodies: The Key to Cutaneous Barrier Function. J. Investig. Dermatol..

[32] Leprince C., Simon M. (2025). Epidermal Lamellar Bodies, Essential Organelles for the Skin Barrier. Front. Cell Dev. Biol..

[33] Mahanty S. (2025). Skin Lamellar Bodies: A Unique Set of Lysosome-Related Organelles. Front. Cell Dev. Biol..

[34] Sugawara T., Iwamoto N., Akashi M., Kojima T., Hisatsune J., Sugai M., Furuse M. (2013). Tight Junction Dysfunction in the Stratum Granulosum Leads to Aberrant Stratum Corneum Barrier Function in Claudin-1-Deficient Mice. J. Dermatol. Sci..

[35] Le L.N., Luu Q.Q., Pham D. (2026). Le Newborn Transepidermal Water Loss and Gestational Age as Predictive Factors for Infant Atopic Dermatitis in the First Three Months of Age. Allergy Asthma Immunol. Res..

[36] Andrew P.V., Pinnock A., Poyner A., Brown K., Chittock J., Kay L.J., Cork M.J., Danby S.G. (2024). Maintenance of an Acidic Skin Surface with a Novel Zinc Lactobionate Emollient Preparation Improves Skin Barrier Function in Patients with Atopic Dermatitis. Dermatol. Ther..

[37] Pretel-Lara C., Sanabria-de la Torre R., Arias-Santiago S., Montero-Vilchez T. (2024). Skin Barrier Function and Microtopography in Patients with Atopic Dermatitis. J. Clin. Med..

[38] Gwon D., Choi H.K., Lee E.O., Hong S.K., Park C.S., Kim J.W., Liu K. (2026). Impact of Ceramide Acyl Chain Length on Human Skin Barrier Recovery and Hydration. J. Cosmet. Dermatol..

[39] Blaess M., Csuk R., Schätzl T., Deigner H.-P. (2024). Elongation of Very Long-Chain Fatty Acids (ELOVL) in Atopic Dermatitis and the Cutaneous Adverse Effect AGEP of Drugs. Int. J. Mol. Sci..

[40] Sassa T., Kihara A. (2014). Metabolism of Very Long-Chain Fatty Acids: Genes and Pathophysiology. Biomol. Ther..

[41] Kawana M., Miyamoto M., Ohno Y., Kihara A. (2020). Comparative Profiling and Comprehensive Quantification of Stratum Corneum Ceramides in Humans and Mice by LC/MS/MS. J. Lipid Res..

[42] Uche L.E., Gooris G.S., Bouwstra J.A., Beddoes C.M. (2021). Increased Levels of Short-Chain Ceramides Modify the Lipid Organization and Reduce the Lipid Barrier of Skin Model Membranes. Langmuir.

[43] Janssens M., van Smeden J., Gooris G.S., Bras W., Portale G., Caspers P.J., Vreeken R.J., Hankemeier T., Kezic S., Wolterbeek R. (2012). Increase in Short-Chain Ceramides Correlates with an Altered Lipid Organization and Decreased Barrier Function in Atopic Eczema Patients. J. Lipid Res..

[44] Shamaprasad P., Nădăban A., Iacovella C.R., Gooris G.S., Bunge A.L., Bouwstra J.A., McCabe C. (2024). The Phase Behavior of Skin-Barrier Lipids: A Combined Approach of Experiments and Simulations. Biophys. J..

[45] Janssens M., van Smeden J., Puppels G.J., Lavrijsen A.P.M., Caspers P.J., Bouwstra J.A. (2014). Lipid to Protein Ratio Plays an Important Role in the Skin Barrier Function in Patients with Atopic Eczema. Br. J. Dermatol..

[46] van Smeden J., Janssens M., Kaye E.C.J., Caspers P.J., Lavrijsen A.P., Vreeken R.J., Bouwstra J.A. (2014). The Importance of Free Fatty Acid Chain Length for the Skin Barrier Function in Atopic Eczema Patients. Exp. Dermatol..

[47] Mojumdar E.H., Kariman Z., van Kerckhove L., Gooris G.S., Bouwstra J.A. (2014). The Role of Ceramide Chain Length Distribution on the Barrier Properties of the Skin Lipid Membranes. Biochim. Biophys. Acta (BBA) -Biomembr..

[48] Chiricozzi A., Maurelli M., Peris K., Girolomoni G. (2020). Targeting IL-4 for the Treatment of Atopic Dermatitis. Immunotargets Ther..

[49] Tsoi L.C., Rodriguez E., Degenhardt F., Baurecht H., Wehkamp U., Volks N., Szymczak S., Swindell W.R., Sarkar M.K., Raja K. (2019). Atopic Dermatitis Is an IL-13–Dominant Disease with Greater Molecular Heterogeneity Compared to Psoriasis. J. Investig. Dermatol..

[50] Zhou J., Gemperline D.C., Turner M.J., Oldach J., Molignano J., Sims J.T., Stayrook K.R. (2021). Transcriptomic Analysis of Healthy and Atopic Dermatitis Samples Reveals the Role of IL-37 in Human Skin. Immunohorizons.

[51] Deleuran M., Thaçi D., Beck L.A., de Bruin-Weller M., Blauvelt A., Forman S., Bissonnette R., Reich K., Soong W., Hussain I. (2020). Dupilumab Shows Long-Term Safety and Efficacy in Patients with Moderate to Severe Atopic Dermatitis Enrolled in a Phase 3 Open-Label Extension Study. J. Am. Acad. Dermatol..

[52] Andrzejczak K., Sternak A., Witkowski W., Ponikowska M. (2025). Inflammation-Driven Molecular Ageing in Chronic Inflammatory Skin Diseases: Is There a Role for Biologic Therapies?. Cells.

[53] Pappa G., Sgouros D., Theodoropoulos K., Kanelleas A., Bozi E., Gregoriou S., Krasagakis K., Katoulis A.C. (2022). The IL-4/-13 Axis and Its Blocking in the Treatment of Atopic Dermatitis. J. Clin. Med..

[54] Czarnowicki T., Gonzalez J., Shemer A., Malajian D., Xu H., Zheng X., Khattri S., Gilleaudeau P., Sullivan-Whalen M., Suárez-Fariñas M. (2015). Severe Atopic Dermatitis Is Characterized by Selective Expansion of Circulating TH2/TC2 and TH22/TC22, but Not TH17/TC17, Cells within the Skin-Homing T-Cell Population. J. Allergy Clin. Immunol..

[55] Yue C., Zhou H., Wang X., Yu J., Hu Y., Zhou P., Zhao F., Zeng F., Li G., Li Y. (2024). Atopic Dermatitis: Pathogenesis and Therapeutic Intervention. MedComm.

[56] Leung D.Y.M., Guttman-Yassky E. (2014). Deciphering the Complexities of Atopic Dermatitis: Shifting Paradigms in Treatment Approaches. J. Allergy Clin. Immunol..

[57] Ong P.Y. (2022). Atopic Dermatitis: Is Innate or Adaptive Immunity in Control? A Clinical Perspective. Front. Immunol..

[58] Furue M. (2020). Regulation of Filaggrin, Loricrin, and Involucrin by IL-4, IL-13, IL-17A, IL-22, AHR, and NRF2: Pathogenic Implications in Atopic Dermatitis. Int. J. Mol. Sci..

[59] Sakai T. (2025). Stratum Corneum Ceramide Abnormalities in Atopic Dermatitis: Pathophysiology and Implications for Disease Management. J. Dermatol..

[60] Flori E., Cavallo A., Mosca S., Kovacs D., Cota C., Zaccarini M., Di Nardo A., Bottillo G., Maiellaro M., Camera E. (2024). JAK/STAT Inhibition Normalizes Lipid Composition in 3D Human Epidermal Equivalents Challenged with Th2 Cytokines. Cells.

[61] Chapman S., Kwa M., Gold L.S., Lim H.W. (2022). Janus Kinase Inhibitors in Dermatology: Part I. A Comprehensive Review. J. Am. Acad. Dermatol..

[62] Huang I.-H., Chung W.-H., Wu P.-C., Chen C.-B. (2022). JAK–STAT Signaling Pathway in the Pathogenesis of Atopic Dermatitis: An Updated Review. Front. Immunol..

[63] Furue M. (2020). Regulation of Skin Barrier Function via Competition between AHR Axis versus IL-13/IL-4-JAK-STAT6/STAT3 Axis: Pathogenic and Therapeutic Implications in Atopic Dermatitis. J. Clin. Med..

[64] Alvarenga J.M., Bieber T., Torres T. (2024). Emerging Biologic Therapies for the Treatment of Atopic Dermatitis. Drugs.

[65] Furue M., Ulzii D., Nakahara T., Tsuji G., Furue K., Hashimoto-Hachiya A., Kido-Nakahara M. (2020). Implications of IL-13Rα2 in Atopic Skin Inflammation. Allergol. Int..

[66] Ulzii D., Kido-Nakahara M., Nakahara T., Tsuji G., Furue K., Hashimoto-Hachiya A., Furue M. (2019). Scratching Counteracts IL-13 Signaling by Upregulating the Decoy Receptor IL-13Rα2 in Keratinocytes. Int. J. Mol. Sci..

[67] Junttila I.S. (2018). Tuning the Cytokine Responses: An Update on Interleukin (IL)-4 and IL-13 Receptor Complexes. Front. Immunol..

[68] Fichtner-Feigl S., Strober W., Kawakami K., Puri R.K., Kitani A. (2006). IL-13 Signaling through the IL-13α2 Receptor Is Involved in Induction of TGF-Β1 Production and Fibrosis. Nat. Med..

[69] Amano W., Nakajima S., Kunugi H., Numata Y., Kitoh A., Egawa G., Dainichi T., Honda T., Otsuka A., Kimoto Y. (2015). The Janus Kinase Inhibitor JTE-052 Improves Skin Barrier Function through Suppressing Signal Transducer and Activator of Transcription 3 Signaling. J. Allergy Clin. Immunol..

[70] Ewald D.A., Malajian D., Krueger J.G., Workman C.T., Wang T., Tian S., Litman T., Guttman-Yassky E., Suárez-Fariñas M. (2015). Meta-Analysis Derived Atopic Dermatitis (MADAD) Transcriptome Defines a Robust AD Signature Highlighting the Involvement of Atherosclerosis and Lipid Metabolism Pathways. BMC Med. Genom..

[71] Danso M., Boiten W., van Drongelen V., Gmelig Meijling K., Gooris G., El Ghalbzouri A., Absalah S., Vreeken R., Kezic S., van Smeden J. (2017). Altered Expression of Epidermal Lipid Bio-Synthesis Enzymes in Atopic Dermatitis Skin Is Accompanied by Changes in Stratum Corneum Lipid Composition. J. Dermatol. Sci..

[72] Toncic R.J., Jakasa I., Hadzavdic S.L., Goorden S.M., van der Vlugt K.J.G., Stet F.S., Balic A., Petkovic M., Pavicic B., Zuzul K. (2020). Altered Levels of Sphingosine, Sphinganine and Their Ceramides in Atopic Dermatitis Are Related to Skin Barrier Function, Disease Severity and Local Cytokine Milieu. Int. J. Mol. Sci..

[73] Ito S., Ishikawa J., Naoe A., Yoshida H., Hachiya A., Fujimura T., Kitahara T., Takema Y. (2017). Ceramide Synthase 4 Is Highly Expressed in Involved Skin of Patients with Atopic Dermatitis. J. Eur. Acad. Dermatol. Venereol..

[74] Das P., Mounika P., Yellurkar M.L., Prasanna V.S., Sarkar S., Velayutham R., Arumugam S. (2022). Keratinocytes: An Enigmatic Factor in Atopic Dermatitis. Cells.

[75] Simmons J., Gallo R.L. (2024). The Central Roles of Keratinocytes in Coordinating Skin Immunity. J. Investig. Dermatol..

[76] Stanbery A.G., Smita S., von Moltke J., Tait Wojno E.D., Ziegler S.F. (2022). TSLP, IL-33, and IL-25: Not Just for Allergy and Helminth Infection. J. Allergy Clin. Immunol..

[77] Torres T., Mendes-Bastos P., Cruz M.J., Duarte B., Filipe P., Lopes M.J.P., Gonçalo M. (2025). Interleukin-4 and Atopic Dermatitis: Why Does It Matter? A Narrative Review. Dermatol. Ther..

[78] Mao-Qiang M., Elias P.M., Feingold K.R. (1993). Fatty Acids Are Required for Epidermal Permeability Barrier Function. J. Clin. Investig..

[79] Uchida Y., Holleran W.M. (2008). Omega-O-Acylceramide, a Lipid Essential for Mammalian Survival. J. Dermatol. Sci..

[80] Elias P.M., Gruber R., Crumrine D., Menon G., Williams M.L., Wakefield J.S., Holleran W.M., Uchida Y. (2014). Formation and Functions of the Corneocyte Lipid Envelope (CLE). Biochim. Biophys. Acta (BBA) Mol. Cell Biol. Lipids.

[81] Bouwstra J.A., Ponec M. (2006). The Skin Barrier in Healthy and Diseased State. Biochim. Biophys. Acta (BBA) -Biomembr..

[82] Jennemann R., Rabionet M., Gorgas K., Epstein S., Dalpke A., Rothermel U., Bayerle A., van der Hoeven F., Imgrund S., Kirsch J. (2012). Loss of Ceramide Synthase 3 Causes Lethal Skin Barrier Disruption. Hum. Mol. Genet..

[83] Fujii M. (2021). The Pathogenic and Therapeutic Implications of Ceramide Abnormalities in Atopic Dermatitis. Cells.

[84] Levy M., Futerman A.H. (2010). Mammalian Ceramide Synthases. IUBMB Life.

[85] Akiyama M. (2014). The Roles of ABCA12 in Epidermal Lipid Barrier Formation and Keratinocyte Differentiation. Biochim. Biophys. Acta (BBA) Mol. Cell Biol. Lipids.

[86] Jiang Y.J., Lu B., Kim P., Paragh G., Schmitz G., Elias P.M., Feingold K.R. (2008). PPAR and LXR Activators Regulate ABCA12 Expression in Human Keratinocytes. J. Investig. Dermatol..

[87] Fischer C.L., Drake D.R., Dawson D.V., Blanchette D.R., Brogden K.A., Wertz P.W. (2012). Antibacterial Activity of Sphingoid Bases and Fatty Acids against Gram-Positive and Gram-Negative Bacteria. Antimicrob. Agents Chemother..

[88] Chieosilapatham P., Kiatsurayanon C., Umehara Y., Trujillo-Paez J.V., Peng G., Yue H., Nguyen L.T.H., Niyonsaba F. (2021). Keratinocytes: Innate Immune Cells in Atopic Dermatitis. Clin. Exp. Immunol..

[89] Akdis C.A., Akdis M., Bieber T., Bindslev-Jensen C., Boguniewicz M., Eigenmann P., Hamid Q., Kapp A., Leung D.Y.M., Lipozencic J. (2006). Diagnosis and Treatment of Atopic Dermatitis in Children and Adults: European Academy of Allergology and Clinical Immunology/American Academy of Allergy, Asthma and Immunology/PRACTALL Consensus Report. J. Allergy Clin. Immunol..

[90] Ricardo-Gonzalez R.R., Red Eagle A., Odegaard J.I., Jouihan H., Morel C.R., Heredia J.E., Mukundan L., Wu D., Locksley R.M., Chawla A. (2010). IL-4/STAT6 Immune Axis Regulates Peripheral Nutrient Metabolism and Insulin Sensitivity. Proc. Natl. Acad. Sci. USA.

[91] Dubrac S., Schmuth M. (2011). PPAR-Alpha in Cutaneous Inflammation. Derm.-Endocrinol..

[92] Yokoyama A., Makishima M., Choi M., Cho Y., Nishida S., Hashimoto Y., Terui T. (2009). Induction of SREBP-1c MRNA by Differentiation and LXR Ligand in Human Keratinocytes. J. Investig. Dermatol..

[93] Moreiras-Arias N., Nieto-Fontarigo J.J., Salgado F.J., González-Vilas D., Paredes-Suárez C., Combo-García E., Rodríguez-Otero C., Flórez Á. (2026). Novel Therapeutic Strategies for Atopic Dermatitis: Biomarker Modulation and Clinical Implications. A Systematic Review. Clin. Rev. Allergy Immunol..

[94] Cole C., Kroboth K., Schurch N.J., Sandilands A., Sherstnev A., O’Regan G.M., Watson R.M., Irwin McLean W.H., Barton G.J., Irvine A.D. (2014). Filaggrin-Stratified Transcriptomic Analysis of Pediatric Skin Identifies Mechanistic Pathways in Patients with Atopic Dermatitis. J. Allergy Clin. Immunol..

[95] Bratu D., Boda D., Caruntu C. (2023). Genomic, Epigenomic, Transcriptomic, Proteomic and Metabolomic Approaches in Atopic Dermatitis. Curr. Issues Mol. Biol..

[96] Huang Y., Chen G., Liu X., Shao Y., Gao P., Xin C., Cui Z., Zhao X., Xu G. (2014). Serum Metabolomics Study and Eicosanoid Analysis of Childhood Atopic Dermatitis Based on Liquid Chromatography–Mass Spectrometry. J. Proteome Res..

[97] Goleva E., Berdyshev E., Kreimer S., Reisz J.A., D’Alessandro A., Bronova I., Lyubchenko T., Richers B.N., Hall C.F., Xiao O. (2025). Longitudinal Integrated Proteomic and Metabolomic Skin Changes in Patients with Atopic Dermatitis Treated with Dupilumab. J. Allergy Clin. Immunol..

[98] Berdyshev E., Goleva E., Bissonnette R., Bronova I., Bronoff A.S., Richers B.N., Garcia S., Ramirez-Gama M., Taylor P., Praestgaard A. (2022). Dupilumab Significantly Improves Skin Barrier Function in Patients with Moderate-to-severe Atopic Dermatitis. Allergy.

[99] Elias P.M., Wakefield J.S. (2014). Mechanisms of Abnormal Lamellar Body Secretion and the Dysfunctional Skin Barrier in Patients with Atopic Dermatitis. J. Allergy Clin. Immunol..

[100] Moosbrugger-Martinz V., Leprince C., Méchin M.-C., Simon M., Blunder S., Gruber R., Dubrac S. (2022). Revisiting the Roles of Filaggrin in Atopic Dermatitis. Int. J. Mol. Sci..

[101] Rerknimitr P., Otsuka A., Nakashima C., Kabashima K. (2017). The Etiopathogenesis of Atopic Dermatitis: Barrier Disruption, Immunological Derangement, and Pruritus. Inflamm. Regen..

[102] Hansen-Sackey E.B., Hartono S. (2025). Atopic Dermatitis: Pathophysiology and Emerging Treatments. Allergies.

[103] Bhattacharya N., Sato W.J., Kelly A., Ganguli-Indra G., Indra A.K. (2019). Epidermal Lipids: Key Mediators of Atopic Dermatitis Pathogenesis. Trends Mol. Med..

[104] Kolb L., Villarreal L.A., Sathe N.C. (2026). Atopic Dermatitis. StatPearls [Internet].

[105] Weidinger S., Novak N. (2016). Atopic Dermatitis. Lancet.

[106] Saleem M.D., Oussedik E., D’Amber V., Feldman S.R. (2017). Interleukin-31 Pathway and Its Role in Atopic Dermatitis: A Systematic Review. J. Dermatol. Treat..

[107] Feld M., Garcia R., Buddenkotte J., Katayama S., Lewis K., Muirhead G., Hevezi P., Plesser K., Schrumpf H., Krjutskov K. (2016). The Pruritus- and TH2-Associated Cytokine IL-31 Promotes Growth of Sensory Nerves. J. Allergy Clin. Immunol..

[108] Yosipovitch G., Berger T., Fassett M.S. (2020). Neuroimmune Interactions in Chronic Itch of Atopic Dermatitis. J. Eur. Acad. Dermatol. Venereol..

[109] Blicharz L., Rudnicka L., Czuwara J., Waśkiel-Burnat A., Goldust M., Olszewska M., Samochocki Z. (2021). The Influence of Microbiome Dysbiosis and Bacterial Biofilms on Epidermal Barrier Function in Atopic Dermatitis—An Update. Int. J. Mol. Sci..

[110] Brunner P.M., Leung D.Y.M., Guttman-Yassky E. (2018). Immunologic, Microbial, and Epithelial Interactions in Atopic Dermatitis. Ann. Allergy Asthma Immunol..

[111] Zheng Y., Hunt R.L., Villaruz A.E., Fisher E.L., Liu R., Liu Q., Cheung G.Y.C., Li M., Otto M. (2022). Commensal Staphylococcus Epidermidis Contributes to Skin Barrier Homeostasis by Generating Protective Ceramides. Cell Host Microbe.

[112] Bibel D.J., Aly R., Shah S., Shinefield H.R. (1993). Sphingosines: Antimicrobial Barriers of the Skin. Acta Derm. Venereol..

[113] Hrestak D., Matijašić M., Čipčić Paljetak H., Ledić Drvar D., Ljubojević Hadžavdić S., Perić M. (2022). Skin Microbiota in Atopic Dermatitis. Int. J. Mol. Sci..

[114] Zhong L., Zhou X., Su J., Zhang Y., Zhang D., Wan H. (2026). Microbiome Dysbiosis and Therapeutic Restoration in Atopic Dermatitis. Front. Cell. Infect. Microbiol..

[115] Lee H.-J., Kim M. (2022). Skin Barrier Function and the Microbiome. Int. J. Mol. Sci..

[116] Zhang X.-E., Zheng P., Ye S.-Z., Ma X., Liu E., Pang Y.-B., He Q.-Y., Zhang Y.-X., Li W.-Q., Zeng J.-H. (2024). Microbiome: Role in Inflammatory Skin Diseases. J. Inflamm. Res..

[117] Andrew P.V., Williams S.F., Brown K., Chittock J., Pinnock A., Poyner A., Cork M.J., Danby S.G. (2025). Topical Supplementation with Physiological Lipids Rebalances the Stratum Corneum Ceramide Profile and Strengthens Skin Barrier Function in Adults Predisposed to Atopic Dermatitis. Br. J. Dermatol..

[118] Nădăban A., Rousel J., El Yachioui D., Gooris G.S., Beddoes C.M., Dalgliesh R.M., Malfois M., Rissmann R., Bouwstra J.A. (2023). Effect of Sphingosine and Phytosphingosine Ceramide Ratio on Lipid Arrangement and Barrier Function in Skin Lipid Models. J. Lipid Res..

[119] Berkers T., Visscher D., Gooris G.S., Bouwstra J.A. (2018). Topically Applied Ceramides Interact with the Stratum Corneum Lipid Matrix in Compromised Ex Vivo Skin. Pharm. Res..

[120] Elias P.M. (2022). Optimizing Emollient Therapy for Skin Barrier Repair in Atopic Dermatitis. Ann. Allergy Asthma Immunol..

[121] Sidbury R., Alikhan A., Bercovitch L., Cohen D.E., Darr J.M., Drucker A.M., Eichenfield L.F., Frazer-Green L., Paller A.S., Schwarzenberger K. (2023). Executive Summary: American Academy of Dermatology Guidelines of Care for the Management of Atopic Dermatitis in Adults with Topical Therapies. J. Am. Acad. Dermatol..

[122] Sidbury R., Alikhan A., Bercovitch L., Cohen D.E., Darr J.M., Drucker A.M., Eichenfield L.F., Frazer-Green L., Paller A.S., Schwarzenberger K. (2023). Guidelines of Care for the Management of Atopic Dermatitis in Adults with Topical Therapies. J. Am. Acad. Dermatol..

[123] van Zuuren E.J., Fedorowicz Z., Christensen R., Lavrijsen A.P., Arents B.W. (2017). Emollients and Moisturisers for Eczema. Cochrane Database Syst. Rev..

[124] Chen X. (2025). Skin Barrier Repair and Nursing Care in Patients with Atopic Dermatitis: A Narrative Review. Int. J. Gen. Med..

[125] Ahlström M.G., Bjerre R.D., Ahlström M.G., Skov L., Johansen J.D. (2024). Stratum Corneum Lipids in Non-Lesional Atopic and Healthy Skin Following Moisturizer Application: A Randomized Clinical Experiment. Life.

[126] Bárány M.L.E. (2000). Skin-Identical Lipids Versus Petrolatum in the Treatment of Tape-Stripped and Detergent-Perturbed Human Skin. Acta Derm. Venereol..

[127] Feingold K.R. (2007). Thematic Review Series: Skin Lipids. The Role of Epidermal Lipids in Cutaneous Permeability Barrier Homeostasis. J. Lipid Res..

[128] Danby S.G., Andrew P.V., Kay L.J., Pinnock A., Chittock J., Brown K., Williams S.F., Cork M.J. (2022). Enhancement of Stratum Corneum Lipid Structure Improves Skin Barrier Function and Protects against Irritation in Adults with Dry, Eczema-prone Skin. Br. J. Dermatol..

[129] Schmuth M., Eckmann S., Moosbrugger-Martinz V., Ortner-Tobider D., Blunder S., Trafoier T., Gruber R., Elias P.M. (2024). Skin Barrier in Atopic Dermatitis. J. Investig. Dermatol..

[130] Gandhi N.A., Bennett B.L., Graham N.M.H., Pirozzi G., Stahl N., Yancopoulos G.D. (2016). Targeting Key Proximal Drivers of Type 2 Inflammation in Disease. Nat. Rev. Drug Discov..

[131] Chatzigeorgiou I., Koumaki D., Vakirlis E., Papadimitriou I., Gregoriou S. (2024). Restoration of Skin Barrier Abnormalities with IL4/13 Inhibitors and Jak Inhibitors in Atopic Dermatitis: A Systematic Review. Medicina.

[132] Levine J., Mehta A., O’Hagan R., Guttman-Yassky E., Lamb A.J. (2026). Decision-Making Factors for Systemic Therapies in Atopic Dermatitis: A Clinical Review. Dermatitis.

[133] de Bruin-Weller M.S., Boesjes C.M., Achten R.A., Beck L.A., Irvine A.D., Vestergaard C., de Graaf M., van Wijk F., Bakker D.S., Weidinger S. (2026). Biologics to Treat Atopic Dermatitis: Effectiveness, Safety, and Future Directions. Allergy.

[134] Boesjes C.M., Kamphuis E., de Graaf M., Spekhorst L.S., Haeck I., van der Gang L.F., Loman L., Zuithoff N.P.A., Dekkers C., van der Rijst L.P. (2024). Long-Term Effectiveness and Reasons for Discontinuation of Dupilumab in Patients with Atopic Dermatitis. JAMA Dermatol..

[135] Halling A.-S., Loft N., Silverberg J.I., Guttman-Yassky E., Thyssen J.P. (2021). Real-World Evidence of Dupilumab Efficacy and Risk of Adverse Events: A Systematic Review and Meta-Analysis. J. Am. Acad. Dermatol..

[136] Dekkers C., Zuithoff N., Bakker D., Knol E., Wevers A., Touwslager W., Christoffers W., Prosje P., van Lynden-van Nes A., van Lümig P. (2025). Tralokinumab Treatment in Adult Atopic Dermatitis Patients: 28-Week Evaluation of Clinical Effectiveness, Safety, Serum Proteins and Total IgE Levels. Allergy.

[137] Beck L.A., Deleuran M., Bissonnette R., de Bruin-Weller M., Galus R., Nakahara T., Seo S.J., Khokhar F.A., Vakil J., Xiao J. (2022). Dupilumab Provides Acceptable Safety and Sustained Efficacy for up to 4 Years in an Open-Label Study of Adults with Moderate-to-Severe Atopic Dermatitis. Am. J. Clin. Dermatol..

[138] Simpson E.L., Gooderham M., Wollenberg A., Weidinger S., Armstrong A., Soung J., Ferrucci S., Lima R.G., Witte M.M., Xu W. (2023). Efficacy and Safety of Lebrikizumab in Combination with Topical Corticosteroids in Adolescents and Adults with Moderate-to-Severe Atopic Dermatitis. JAMA Dermatol..

[139] Simpson E.L., Pink A.E., Blauvelt A., Gooderham M., Armstrong A.W., Worm M., Katoh N., Peris K., Puig L., Barbarot S. (2023). Tralokinumab Efficacy Over 1 Year in Adults with Moderate-to-Severe Atopic Dermatitis: Pooled Data from Two Phase III Trials. Am. J. Clin. Dermatol..

[140] Guttman-Yassky E., Blauvelt A., Eichenfield L.F., Paller A.S., Armstrong A.W., Drew J., Gopalan R., Simpson E.L. (2020). Efficacy and Safety of Lebrikizumab, a High-Affinity Interleukin 13 Inhibitor, in Adults with Moderate to Severe Atopic Dermatitis. JAMA Dermatol..

[141] Le Floc’h A., Allinne J., Nagashima K., Scott G., Birchard D., Asrat S., Bai Y., Lim W.K., Martin J., Huang T. (2020). Dual Blockade of IL-4 and IL-13 with Dupilumab, an IL-4Rα Antibody, Is Required to Broadly Inhibit Type 2 Inflammation. Allergy.

[142] Gonçalves F., Freitas E., Torres T. (2021). Selective IL-13 Inhibitors for the Treatment of Atopic Dermatitis. Drugs Context.

[143] Lytvyn Y., Gooderham M. (2023). Targeting Interleukin 13 for the Treatment of Atopic Dermatitis. Pharmaceutics.

[144] Ahn J., Choi Y., Simpson E.L. (2021). Therapeutic New Era for Atopic Dermatitis: Part 1. Biologics. Ann. Dermatol..

[145] Sander N., Stölzl D., Fonfara M., Hartmann J., Harder I., Suhrkamp I., Jakaša I., van den Bogaard E., van Vlijmen-Willems I., Szymczak S. (2024). Blockade of Interleukin-13 Signalling Improves Skin Barrier Function and Biology in Patients with Moderate-to-Severe Atopic Dermatitis. Br. J. Dermatol..

[146] Guttman-Yassky E., Sun Z., Mena L.R., Hahn N., Nickoloff B.J., Preuss C., Siu K., Natalie C.R., Gallo G., Wolf E. (2025). Lebrikizumab Rapidly Lowers Inflammatory Biomarkers with Clinical Correlations in Moderate-to-Severe Atopic Dermatitis. Dermatol. Ther..

[147] Cevikbas F., Ward A., Veverka K.A. (2024). Eblasakimab, an Anti-IL-13Rα1 Antibody, Reduces Atopy-Associated Serum Biomarkers in Moderate-to-Severe Atopic Dermatitis. BioDrugs.

[148] Cevikbas F., Ward A., Firth C., Veverka K. (2023). Eblasakimab, a Novel IL-13 Receptor Alpha 1 Monoclonal Antibody, Blocks STAT6 Phosphorylation with Low Dose in Human Volunteers. Clin. Immunol..

[149] Veverka K.A., Thng S.T.G., Silverberg J.I., Armstrong A.W., Menezes J., Kaoukhov A., Blauvelt A. (2024). Safety and Efficacy of Eblasakimab, an Interleukin 13 Receptor A1 Monoclonal Antibody, in Adults with Moderate-to-Severe Atopic Dermatitis: A Phase 1b, Multiple-Ascending Dose Study. J. Am. Acad. Dermatol..

[150] Montero-Vilchez T., Rodriguez-Pozo J.-A., Diaz-Calvillo P., Salazar-Nievas M., Tercedor-Sanchez J., Molina-Leyva A., Arias-Santiago S. (2022). Dupilumab Improves Skin Barrier Function in Adults with Atopic Dermatitis: A Prospective Observational Study. J. Clin. Med..

[151] Cork M.J., Berdyshev E., Goleva E., Danby S.G., Bronova I., Zahn J., Agueusop I., Bafna S., Ong P., Zhang A. (2024). 52273 Dupilumab Provides Sustained Normalization of Skin Barrier Function in Children with Moderate-to-Severe Atopic Dermatitis. J. Am. Acad. Dermatol..

[152] Zhang L., Wen X., Hou Y., Yang Y., Song W., Zeng Y., Sun J. (2022). Integrated Metabolomics and Lipidomics Study of Patients with Atopic Dermatitis in Response to Dupilumab. Front. Immunol..

[153] Goleva E., Calatroni A., LeBeau P., Berdyshev E., Taylor P., Kreimer S., Cole R.N., Leung D.Y.M. (2020). Skin Tape Proteomics Identifies Pathways Associated with Transepidermal Water Loss and Allergen Polysensitization in Atopic Dermatitis. J. Allergy Clin. Immunol..

[154] Nakashima C., Yanagihara S., Otsuka A. (2022). Innovation in the Treatment of Atopic Dermatitis: Emerging Topical and Oral Janus Kinase Inhibitors. Allergol. Int..

[155] Rodrigues M.A., Torres T. (2020). JAK/STAT Inhibitors for the Treatment of Atopic Dermatitis. J. Dermatol. Treat..

[156] Tsiogka A., Kyriazopoulou M., Kontochristopoulos G., Nicolaidou E., Stratigos A., Rigopoulos D., Gregoriou S. (2022). The JAK/STAT Pathway and Its Selective Inhibition in the Treatment of Atopic Dermatitis: A Systematic Review. J. Clin. Med..

[157] Solimani F., Meier K., Ghoreschi K. (2019). Emerging Topical and Systemic JAK Inhibitors in Dermatology. Front. Immunol..

[158] Kim Y., Seo G., Koopman J.J.E., Yee J. (2025). Real-World Effectiveness and Safety of JAK Inhibitors in Atopic Dermatitis: A Systematic Review and Meta-Analysis. Clin. Exp. Allergy.

[159] Guttman-Yassky E., Silverberg J.I., Nemoto O., Forman S.B., Wilke A., Prescilla R., de la Peña A., Nunes F.P., Janes J., Gamalo M. (2019). Baricitinib in Adult Patients with Moderate-to-Severe Atopic Dermatitis: A Phase 2 Parallel, Double-Blinded, Randomized Placebo-Controlled Multiple-Dose Study. J. Am. Acad. Dermatol..

[160] Davis D.M.R., Drucker A.M., Alikhan A., Bercovitch L., Cohen D.E., Darr J.M., Eichenfield L.F., Frazer-Green L., Paller A.S., Schwarzenberger K. (2024). Guidelines of Care for the Management of Atopic Dermatitis in Adults with Phototherapy and Systemic Therapies. J. Am. Acad. Dermatol..

[161] Gao Q., Zhao Y., Zhang J. (2023). Efficacy and Safety of Abrocitinib and Upadacitinib versus Dupilumab in Adults with Moderate-to-Severe Atopic Dermatitis: A Systematic Review and Meta-Analysis. Heliyon.

[162] Irvine A.D., Prajapati V.H., Guttman-Yassky E., Simpson E.L., Papp K.A., Blauvelt A., Chu C.-Y., Hong H.C., Gold L.F.S., de Bruin-Weller M. (2025). Efficacy and Safety of Upadacitinib in Patients with Moderate-to-Severe Atopic Dermatitis: Phase 3 Randomized Clinical Trial Results Through 140 Weeks. Am. J. Clin. Dermatol..

[163] Reich K., Thyssen J.P., Blauvelt A., Eyerich K., Soong W., Rice Z.P., Hong H.C., Katoh N., Valenzuela F., DiBonaventura M. (2022). Efficacy and Safety of Abrocitinib versus Dupilumab in Adults with Moderate-to-Severe Atopic Dermatitis: A Randomised, Double-Blind, Multicentre Phase 3 Trial. Lancet.

[164] Silverberg J.I., Simpson E.L., Thyssen J.P., Gooderham M., Chan G., Feeney C., Biswas P., Valdez H., DiBonaventura M., Nduaka C. (2020). Efficacy and Safety of Abrocitinib in Patients with Moderate-to-Severe Atopic Dermatitis. JAMA Dermatol..

[165] Lee K.P., Plante J., Korte J.E., Elston D.M. (2023). Oral Janus Kinase Inhibitors in the Treatment of Atopic Dermatitis: A Systematic Review and Meta-Analysis. Ski. Health Dis..

[166] Kogame T., Egawa G., Kabashima K. (2023). Exploring the Role of Janus Kinase (JAK) in Atopic Dermatitis: A Review of Molecular Mechanisms and Therapeutic Strategies. Immunol. Med..

[167] Chovatiya R., Paller A.S. (2021). JAK Inhibitors in the Treatment of Atopic Dermatitis. J. Allergy Clin. Immunol..

[168] Guttman-Yassky E., Thaçi D., Pangan A.L., Hong H.C., Papp K.A., Reich K., Beck L.A., Mohamed M.-E.F., Othman A.A., Anderson J.K. (2020). Upadacitinib in Adults with Moderate to Severe Atopic Dermatitis: 16-Week Results from a Randomized, Placebo-Controlled Trial. J. Allergy Clin. Immunol..

[169] Blauvelt A., Teixeira H.D., Simpson E.L., Costanzo A., De Bruin-Weller M., Barbarot S., Prajapati V.H., Lio P., Hu X., Wu T. (2021). Efficacy and Safety of Upadacitinib vs Dupilumab in Adults with Moderate-to-Severe Atopic Dermatitis. JAMA Dermatol..

[170] Mikhaylov D., Ungar B., Renert-Yuval Y., Guttman-Yassky E. (2023). Oral Janus Kinase Inhibitors for Atopic Dermatitis. Ann. Allergy Asthma Immunol..

[171] Cork M.J., Ong P.Y., Danby S.G., Byers R.A., Matcher S.J., Katibi O.S., Brown K., Andrew P.V., Pinnock A., Poyner A. (2026). Dupilumab Treatment Restores Epithelial Barrier in Nonlesional and Lesional Skin in Children with Atopic Dermatitis. Ann. Allergy Asthma Immunol..

[172] Cork M.J., Danby S.G., Vasilopoulos Y., Hadgraft J., Lane M.E., Moustafa M., Guy R.H., MacGowan A.L., Tazi-Ahnini R., Ward S.J. (2009). Epidermal Barrier Dysfunction in Atopic Dermatitis. J. Investig. Dermatol..

[173] Elias P.M., Wakefield J.S. (2011). Therapeutic Implications of a Barrier-Based Pathogenesis of Atopic Dermatitis. Clin. Rev. Allergy Immunol..

[174] Paller A.S., Kong H.H., Seed P., Naik S., Scharschmidt T.C., Gallo R.L., Luger T., Irvine A.D. (2019). The Microbiome in Patients with Atopic Dermatitis. J. Allergy Clin. Immunol..

[175] Wollenberg A., Barbarot S., Bieber T., Christen-Zaech S., Deleuran M., Fink-Wagner A., Gieler U., Girolomoni G., Lau S., Muraro A. (2018). Consensus-based European Guidelines for Treatment of Atopic Eczema (Atopic Dermatitis) in Adults and Children: Part I. J. Eur. Acad. Dermatol. Venereol..

[176] Boer D.E.C., van Smeden J., Al-Khakany H., Melnik E., van Dijk R., Absalah S., Vreeken R.J., Haenen C.C.P., Lavrijsen A.P.M., Overkleeft H.S. (2020). Skin of Atopic Dermatitis Patients Shows Disturbed β-Glucocerebrosidase and Acid Sphingomyelinase Activity That Relates to Changes in Stratum Corneum Lipid Composition. Biochim. Biophys. Acta (BBA) Mol. Cell Biol. Lipids.

[177] Chamlin S.L., Kao J., Frieden I.J., Sheu M.Y., Fowler A.J., Fluhr J.W., Williams M.L., Elias P.M. (2002). Ceramide-Dominant Barrier Repair Lipids Alleviate Childhood Atopic Dermatitis: Changes in Barrier Function Provide a Sensitive Indicator of Disease Activity. J. Am. Acad. Dermatol..

[178] Hartmann D., Retamal C., Valenzuela F. (2025). Precision Medicine and Treat-to-Target Approach in Atopic Dermatitis: Enhancing Personalized Care and Outcomes. An. Bras. Dermatol..

[179] Sanabria-de la Torre R., Montero-Vílchez T., García-Gavín J., Arias-Santiago S. (2025). Current Insights on Lipidomics in Dermatology: A Systematic Review. J. Investig. Dermatol..

[180] Renert-Yuval Y., Thyssen J.P., Bissonnette R., Bieber T., Kabashima K., Hijnen D., Guttman-Yassky E. (2021). Biomarkers in Atopic Dermatitis—A Review on Behalf of the International Eczema Council. J. Allergy Clin. Immunol..

[181] Sakai T., Hatano Y. (2025). Stratum Corneum PH and Ceramides: Key Regulators and Biomarkers of Skin Barrier Function in Atopic Dermatitis. J. Dermatol. Sci..

[182] Nakajima S., Nakamizo S., Nomura T., Ishida Y., Sawada Y., Kabashima K. (2024). Integrating Multi-omics Approaches in Deciphering Atopic Dermatitis Pathogenesis and Future Therapeutic Directions. Allergy.

[183] von Gerichten J., Saunders K., Bailey M.J., Gethings L.A., Onoja A., Geifman N., Spick M. (2024). Challenges in Lipidomics Biomarker Identification: Avoiding the Pitfalls and Improving Reproducibility. Metabolites.

[184] Keurentjes A.J., Jakasa I., Kezic S. (2021). Research Techniques Made Simple: Stratum Corneum Tape Stripping. J. Investig. Dermatol..

